# Productive Entry of Foot-and-Mouth Disease Virus via Macropinocytosis Independent of Phosphatidylinositol 3-Kinase

**DOI:** 10.1038/srep19294

**Published:** 2016-01-13

**Authors:** Shi-Chong Han, Hui-Chen Guo, Shi-Qi Sun, Ye Jin, Yan-Quan Wei, Xia Feng, Xue-Ping Yao, Sui-Zhong Cao, Ding Xiang Liu, Xiang-Tao Liu

**Affiliations:** 1 State Key Laboratory of Veterinary Etiological Biology and OIE/National Foot and Mouth Disease Reference Laboratory, Lanzhou Veterinary Research Institute, Chinese Academy of Agricultural Sciences, Lanzhou, Gansu, China; 2 College of Veterinary Medicine, Sichuan Agricultural University, Ya’an, Sichuan, China; 3 School of Biological Sciences, Nanyang Technological University, Singapore

## Abstract

Virus entry is an attractive target for therapeutic intervention. Here, using a combination of electron microscopy, immunofluorescence assay, siRNA interference, specific pharmacological inhibitors, and dominant negative mutation, we demonstrated that the entry of foot-and-mouth disease virus (FMDV) triggered a substantial amount of plasma membrane ruffling. We also found that the internalization of FMDV induced a robust increase in fluid-phase uptake, and virions internalized within macropinosomes colocalized with phase uptake marker dextran. During this stage, the Rac1-Pak1 signaling pathway was activated. After specific inhibition on actin, Na^+^/H^+^ exchanger, receptor tyrosine kinase, Rac1, Pak1, myosin II, and protein kinase C, the entry and infection of FMDV significantly decreased. However, inhibition of phosphatidylinositol 3-kinase (PI3K) did not reduce FMDV internalization but increased the viral entry and infection to a certain extent, implying that FMDV entry did not require PI3K activity. Results showed that internalization of FMDV exhibited the main hallmarks of macropinocytosis. Moreover, intracellular trafficking of FMDV involves EEA1/Rab5-positive vesicles. The present study demonstrated macropinocytosis as another endocytic pathway apart from the clathrin-mediated pathway. The findings greatly expand our understanding of the molecular mechanisms of FMDV entry into cells, as well as provide potential insights into the entry mechanisms of other picornaviruses.

Foot-and-mouth disease virus (FMDV), the prototypic member of the genus *Aphthovirus* within the family *Picornaviridae*, is responsible for an acute, highly contagious, and economically important disease of cloven-hoofed livestock; this disease is characterized by high fever and vesicular lesions on the tongue, lips, teats, nares, and feet^1^,^2^,^3^,^4^. Foot-and-mouth disease (FMD) remains widespread in extensive areas worldwide, particularly in Asia and Africa^4^.

FMDV is non-enveloped and consists of a densely packed icosahedral protein shell, which is approximately 30 nm in diameter surrounding a single-stranded, positive-sense RNA genome of about 8400 nt in length^3^,^4^. This genome can encode a polyprotein followed by proteolytic cleavage to produce a dozen mature polypeptides and various intermediate precursors. Four structural proteins (VP1–VP4), which are encoded by the N-terminal P1 region of the genome, are required for viral assembly and mediate viral immunogenicity and binding to cell receptors. However, nonstructural proteins derived from the P2 and P3 regions participate in proteolytic activity, RNA replication, and virulence^2^,^3^.

A successful entry into cells is the precondition for viral infection. An in-depth study on the endocytic mechanism of virus can provide potential targets for the development of new antiviral drugs and potential vaccines. Most viruses enter the cells by host cell’s endocytic mechanisms, thereby introducing their genetic material to the preferred replication region^5^,^6^. Previous studies have revealed many endocytic pathways for viral entry into cells, of which clathrin-mediated endocytosis (CME) is the most strongly concerned. In CME, clathrin is assembled on the cytoplasmic face of the plasma membrane to form a clathrin-coated pit (CCP). Internalization of the virus-receptor complex occurs in CCP, which then invaginates toward the cytoplasmic side and becomes dissociated from the cell membrane. Thus, clathrin-coated vesicles containing the endocytic cargo are formed with a diameter of about 120 nm, which then deliver the cargo into endosomes. This process depends on adaptor protein 2, GTPase dynamin, and other important accessory proteins, such as AP180 and Eps15^7^,^8^. Viruses using this pathway include Semliki forest virus^9^, vesicular stomatitis virus^10^, and adenovirus 2/5^8^. Another known endocytic pathway is mediated by caveolin with the formation of caveolae, which are flask-shaped invaginations in the plasma membrane. This process depends on caveolin, lipid raft, and cholesterol^7^,^8^. The polyomavirus family, including SV40 and mouse polyomavirus, can use this pathway to enter cells^8^,^11^,^12^.

Many other viruses also utilize the macropinocytosis or macropinocytosis-like pathway to enter cells. Macropinocytosis is different from other pinocytic pathways because after the virus binds to the cell membrane, it activates the receptor tyrosine kinases (RTKs) or other signaling molecules to further activate the intracellular multi-branched signaling cascades. Subsequently, actin rearrangement is triggered, finally leading to the appearance of numerous irregular ruffles and blebs on the cell membrane. These ruffles collapse toward the cell membrane, enveloping the virus-receptor complex together with the dissociated virus and other fluid-phase macromolecules^13^,^14^,^15^,^16^. Finally, they pinch off from the cell membrane and enter the cytoplasm as large (0.5–10 μm) and irregularly shaped endocytic vesicles known as macropinosomes^14^,^15^,^16^. Macropinocytosis involves various proteins, including Rho GTPases (Rac1 or Cdc42), Na^+^/H^+^ exchangers (NHEs), kinases [Pak1, PI3K, or protein kinase C (PKC)], myosin II, and dymanin-2; the requirement for these factors varies with cell or virus types^15^,^17^. Vaccinia virus^13^,^18^, Ebola virus^19^,^20^, influenza virus^21^,^22^, adenovirus type 35^17^ and picornaviruses, such as echovirus 1 (EV1)^23^ and coxsackievirus B (CVB)^24^, enter cells via macropinocytosis. Besides, cellular uptake of human Rhinoviruses 8 and 14 can occur via a pathway exhibiting some characteristics of macropinocytosis^25^,^26^.

Existing studies have shown that the field isolates of FMDV using four different types of RGD-dependent integrin receptors (αvβ1, αvβ3, αvβ6, and αvβ8) enter cells via the CME pathway^27^,^28^,^29^,^30^. Cell culture-adapted isolates can bind to heparan sulfate (HS) to enter the cells by a caveolin-mediated endocytic pathway^31^. Although several picornaviruses, such as EV1, CVB, CVA9 and HRV8/14, can enter the cells by macropinocytosis, whether FMDV can use this pathway is rarely reported. In some studies, other non-integrins or non-HS receptors can also mediate FMDV infection using an as-yet-unidentified mechanism^32^. In the present work, we selected baby hamster kidney (BHK-21) and pig kidney (PK-15) cells as experimental materials to achieve an in-depth analysis of FMDV endocytosis. BHK-21 and PK-15 are conventional cell lines used for FMDV research and vaccine production. By combining optical and electron microscopy, immunofluorescence, specific pharmacological inhibitor treatment, small interfering RNA (siRNA) silencing, and overexpression of dominant-negative (DN) mutant, we showed that FMDV was taken up predominantly by macropinocytosis to a certain degree, with the CME pathway as a supplementary pathway. This study demonstrated a novel pathway for FMDV entry and may facilitate understanding of the endocytic mechanisms of other picornaviruses.

## Results

### Kinetic characteristics of FMDV entry into cells and its replication

**Figure 1:**
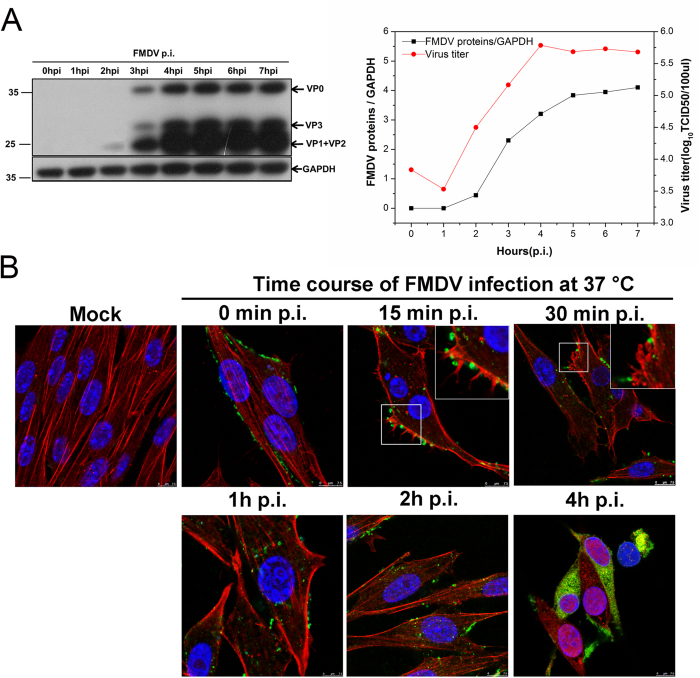
Kinetics of FMDV internalization and replication into BHK-21 cells. **(A)** One-step growth curves of FMDV in BHK-21 cells. FMDV (MOI 1) was bound to BHK-21 cells at 4 °C. Unbound virus was removed, and the cells were transferred to 37 °C. At the indicated time points, the FMDV-infected cells were collected for immunoblotting with an anti-FMDV antibody to determine the amount of FMDV capsid proteins (VP0, VP3, VP1, and VP2). GAPDH was detected as a load control. The relative quantification of the viral proteins was determined by densitometry as shown in the black line of the graph (right). The FMDV-infected cells were removed to −70 °C, and samples were repeatedly frozen and thawed three times. Virus titers were determined by TCID50 on BHK-21 cells as shown in the red line of the graph (right). **(B)** Time course of FMDV infection in BHK-21 cells. Cells were infected with FMDV (MOI 25) at 4 °C. After the adsorption period, the cells were washed with PBS and incubated at 37 °C. Cells were fixed at the indicated time points and processed for CLSM with AF594-phalloidin, anti-FMDV, and DAPI to stain actin filaments (red), viral particles (green), and cell nuclei (blue), respectively.

BHK-21 and PK-15 cells are ideal cell models for research on FMDV and vaccine development. To accurately define the conditions of FMDV internalization and viral replication cycle in cells, Asia I FMDV was used to infect BHK-21 and PK-15 cells. Optical microscope observations revealed that after the BHK-21 cells were infected by FMDV (MOI 1) for 4 h, the cells presented obvious CPE. A longer infection time indicated more evident CPE. At 8 hpi, more cells floated in the culture solution (Supplementary Fig. 1A). Thus, viral replication characteristics within 8 h of viral infection were determined. Western blot assay results showed that viral proteins were detected at 2 h after viral infection, and TCID50 results showed that the virus titer increased rapidly at 2 h after infection. These results above indicated that a 2 h time point possibly showed the earliest time point when capsid proteins and genome RNA were being produced. The amount of capsid proteins and viral titers reached and maintained peak values at 4–5 hpi (Fig. 1A). Localization of FMDV inside BHK-21 cells was observed at indicated time points using CSLM. FMDV (MOI 25) was adsorbed on the cell surface at 4 °C. However, after the cells were shifted to 37 °C for 15 min, some FMDVs were internalized and distributed on the margin of the cells. With prolonged infection time, FMDVs moved toward the nuclei, and a specific green signal was gradually enhanced. At 4 hpi, the viral proteins filled the entire cytoplasm (Fig. 1B). These results above showed that FMDV possibly completed the replication cycle after about 4 hpi. FMDV replication in PK-15 cells presented features similar to those in BHK-21 cells (Supplementary Fig. 1B). Thus, in subsequent experiments, 4 h after FMDV infection was considered the optimal time point for determining the viral titers, viral genes, and protein expression levels.

### CME pathway is not the only pathway for FMDV internalization into the cells

**Figure 2:**
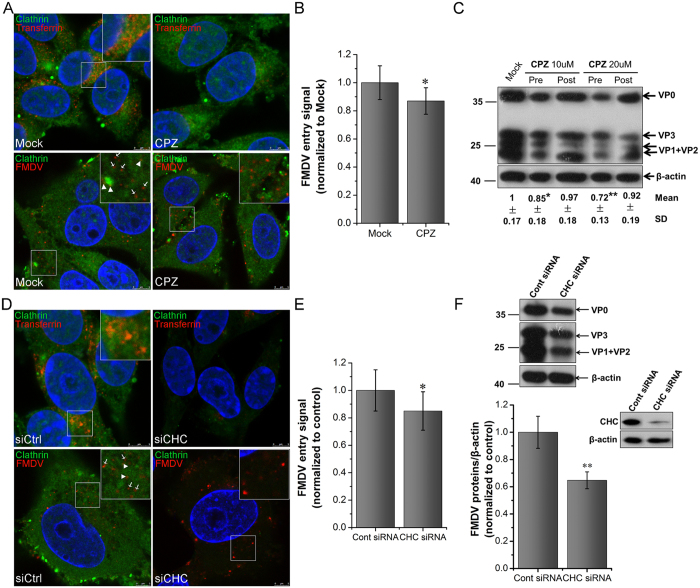
CME is not the only pathway for FMDV internalization into BHK-21. **(A–C)** CPZ moderately inhibited FMDV entry and infection. **(A)** Cells were pretreated with CPZ (20 μM) and maintained during infection. The effect of CPZ on Alexa Fluor 594–TF uptake was apparent (red; upper panels). After 1 hpi (FMDV, MOI 25), cells were fixed and incubated with anti-clathrin, anti-FMDV, and DAPI to stain clathrin (green), viral particles (red), and cell nuclei (blue), respectively (upper panels). **(B)** Quantitative analysis of the internalization of FMDV. The internalized FMDV were analyzed in 10 individual Mock- or CPZ-treated cells. Each experiment was performed in triplicate and the results were presented as the mean ± SD. **(C)** Cells were treated with CPZ at 30 min before the infection (Pre) or treated 60 min after virus addition (Post) and maintained during the infection. After 4 hpi (FMDV, MOI 1), equivalent amounts of protein were analyzed by Western blot with an anti-FMDV antibody, and β-actin was used as the internal control. Fold induction was determined by densitometry. **(D–F)** CHC downregulation moderately inhibited FMDV entry and infection. **(D)** Cells were transfected with control siRNA (left panels) or CHC siRNA to downregulate CHC expression (right panels). The efficiency of CHC downregulation was analyzed by immunofluorescent staining at 36 h post-transfection (green); the effect of siRNA on AF594–TF uptake was apparent (red; upper panels). After 1 hpi (FMDV, MOI 25), siRNA-transfected cells were processed for confocal microscopy as in (**A**). **(E)** Quantitative analysis of the internalization of FMDV in siRNA-transfected cells. **(F)** The efficiency of CHC downregulation was analyzed by immunoblotting. After 4 hpi (FMDV, MOI 1), the siRNA-transfected cells were processed for Western blot. SD, standard deviation; *P < 0.05; **P < 0.01.

Previous studies have demonstrated that FMDV can recognize integrin receptors (αvβ1, αvβ3, αvβ6, and αvβ8) on the cell surface and enter cells via the CME pathway^27^,^28^,^29^,^30^. To assess the role of the CME pathway in Asia I FMDV entry into BHK-21 and PK15 cells, chlorpromazine (CPZ) was used to specifically block this pathway^33^, and the effects on entry and infection of FMDV were assessed. CPZ treatment at 20 μM did not affect cell vitality (Supplementary Fig. 2). Transferrin (TF), the model molecule in the clathrin-dependent pathway, was also used as a positive control. CPZ treatment could significantly reduce the uptake of TF (Fig. 2A), indicating that CPZ could effectively block this pathway. However, during CPZ treatment prior to FMDV addition, FMDV internalization and multiplication were moderately decreased (Fig. 2A–C). Once FMDV entered the cells, CPZ treatment produced no effect on FMDV infection (Fig. 2C). Similar results were obtained in PK-15 cells treated with CPZ (Supplementary Fig. 3A). These findings indicated that CPZ treatment exhibited an inhibitory effect on FMDV uptake but did not influence FMDV proliferation. To further determine the role of clathrin on FMDV internalization, the expression levels of clathrin were downregulated by the specific siCHC. Downregulation of CHC expression (green) was confirmed by immunofluorescent staining and Western blot in BHK-21 cells (Fig. 2D,F). Compared with the control siRNA, CHC siRNA significantly inhibited TF uptake (Fig. 2D). Similar to the results of CPZ treatment, FMDV internalization and multiplication were moderately inhibited after the downregulation of CHC expression (Fig. 2D–F). Thus, the CME pathway may not be the only pathway of FMDV invasion.

CLSM was applied to observe the colocalization of either FMDV or TF with clathrin. FMDV was colocalized only partially with clathrin. By contrast, TF was nearly completely colocalized with clathrin (Fig. 2A). This result further confirmed that during the process of FMDV entry, alternative pathways independent from clathrin may exist.

### FMDV entry does not depend on caveolin but requires plasma membrane cholesterol

**Figure 3:**
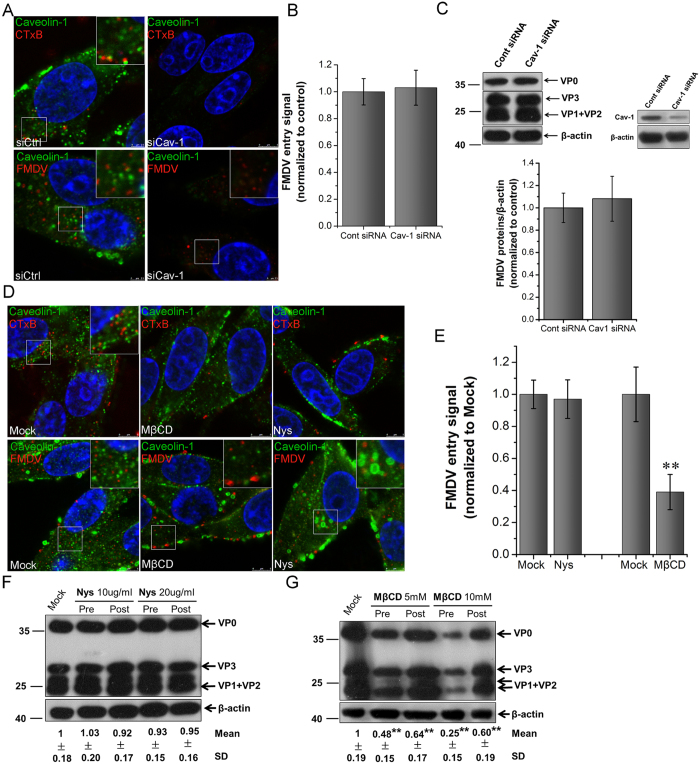
FMDV internalization and replication in BHK-21 cells are Cav-1 independent but require plasma membrane cholesterol. **(A,B)** Cav-1 downregulation did not affect the internalization of FMDV. Cells were transfected with control siRNA (left panels) or Cav-1 siRNA to downregulate Cav-1 expression (right panels). The effect of siRNA on AF594–CTxB uptake was apparent (red; upper panels). FMDV (MOI 25) was allowed to bind to siRNA-transfected cells for 1 h at 4 °C and then transferred to 37 °C. After incubation for 1 h at 37 °C, the fixed cells were processed for confocal microscopy (lower panels). **(B)** Quantitative analysis of the internalization of FMDV in siRNA-transfected cells. The internalized FMDV were analyzed in 10 individual siRNA-transfected cells. Each experiment was performed in triplicate and the results were presented as the mean ± SD. **(C)** Cav-1 downregulation did not affect the synthesis of viral proteins. The siRNA-transfected cells were infected (MOI 1) for 4 h and analyzed with an anti-FMDV antibody in Western blot, and β-actin was measured as the internal control. The relative quantification of the viral proteins was determined by densitometry as shown in the histogram. **(D)** MβCD inhibited the internalization of FMDV, whereas Nys did not. Cells were pretreated with MβCD (10 mM) or Nys (20 μg/mL) and then infected (MOI 25) as described in the Materials and Methods. Samples were then processed for confocal microscopy as in (**A**). **(E)** Quantitative analysis of the internalization of FMDV in Mock-, Nys- or MβCD-treated cells. **(F)** Nys did not affect FMDV entry and replication. Cells were treated with Nys 30 min before the infection (Pre) or treated 60 min after virus addition (Post) and maintained during the infection. After 4 hpi (FMDV, MOI 1) equivalent amounts of protein were analyzed in immunoblots, and fold induction was determined by densitometry. **(G)** MβCD inhibited FMDV entry and multiplication. MβCD was present only during treatment for 30 min before the infection (Pre) or 60 min after virus addition (Post). Samples were then processed for Western blot. SD, standard deviation; *P < 0.05; **P < 0.01.

As the main scaffold protein of caveolae, caveolin-1 (Cav-1) plays a crucial role in the formation and stability of caveolae^7^,^8^. A recent study^31^ reported that FMDV utilizing HS receptors enters cells via the caveolin-mediated endocytic pathway. However, in the present work, the role of the caveolin-mediated endocytic pathway in FMDV internalization was tested by inhibiting Cav-1 expression with siRNA. The silencing efficiency of Cav-1 was confirmed by immunofluorescent staining and Western blot. Cav-1 siRNA treatment significantly inhibited CTxB uptake (Fig. 3A), but showed almost no effect on FMDV internalization and infection (Fig. 3A–C). cholera toxin B (CTxB) utilizing the caveolin-dependent endocytic pathway to enter cells was administered as a positive control^34^. Thus, FMDV entry into cells was independent of the caveolin-mediated endocytic pathway. CLSM was also applied to observe colocalization of either FMDV or CTxB with Cav-1. FMDV was not colocalized with Cav-1 at the early stage of internalization, but most of CTxB was colocalized with Cav-1 (Fig. 3D). This result further confirmed that FMDV entry into cells was independent of the caveolin-mediated endocytic pathway.

The caveolin-mediated endocytic pathway strictly depends on cholesterol, which is the main component of lipid rafts in caveolae^7^,^15^. In the present study, methyl-β-cyclodextrin (MβCD, cholesterol-deleting compound)^35^ and nystatin (Nys, cholesterol-sequestering compound)^36^ of indicated concentrations were used to treat the cells (Supplementary Fig. 2). CLSM images showed that MβCD- and Nys-treated BHK-21 cells obviously inhibited the internalization of CTxB (Fig. 3D). However, Nys treatment before or after virus addition did not influence FMDV infection in BHK-21 (Fig. 3D–F). MβCD treatment before virus addition significantly inhibited the internalization and multiplication of FMDV (Fig. 3D–G). Consistently, if MβCD was administered 60 min after FMDV addition, viral infection was also significantly inhibited (Fig. 3G). This result was consistent with that of Miguel *et al.*^37^. Similar results were obtained in PK-15 cells treated with the two inhibitors (Supplementary Fig. 3B,C). The above results indicated that the disorder of caveolae caused by Nys did not affect FMDV entry. By contrast, cholesterol loss caused by MβCD affected not only the early entry of FMDV but also FMDV multiplication. Hence, FMDV entry into cells did not depend on caveolae but required the involvement of cholesterol.

### FMDV entry depends on actin dynamics and induces actin ruffles

The above experimental results indicated that FMDV entry into cells depended on the CME pathway, but not the caveolin-mediated endocytic pathway. Moreover, the former was not the only pathway for FMDV internalization. The kinetic characteristics of FMDV internalization into BHK-21 cells were observed by CLSM. FMDV entry at the early stage (15 min) induced the change in actin skeleton from fibrous to floccus. However, the actin distribution returned to normal within 2 hpi. Considerable cell-wide ruffling was also observed on the cell membrane (Fig. 1B). These observations agreed well with the distinguishing features of macropinocytosis compared with the endocytic pathways^15^,^16^. Thus, macropinocytosis may be another potential pathway for the productive entry of FMDV.

**Figure 4:**
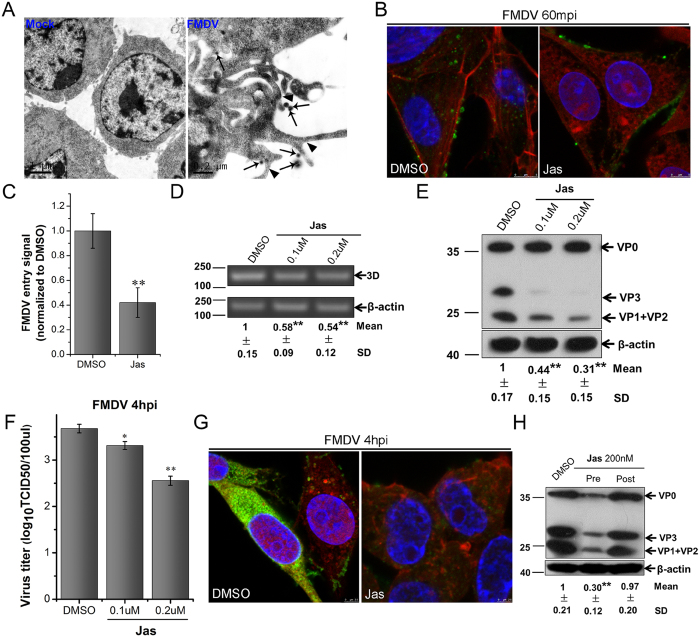
FMDV entry into BHK-21 cells depends on actin dynamics and induces actin ruffles. **(A)** FMDV entry induced actin ruffles. Cells were incubated with FMDV (MOI 100) for 1 h at 37 °C prior to incubation for 5 min at 37 °C, fixed, and processed for TEM. The arrow indicates a virion. The arrowhead indicates the membrane ruffle. **(B,C)** Jas inhibited FMDV entry. Pretreated cells (0.2 μM Jas) were infected (MOI 25) for 1 h at 37 °C and processed for confocal microscopy with AF594-phalloidin (red), anti-FMDV (green), and DAPI (blue). **(C)** Quantitative analysis of the internalization of FMDV. The internalized FMDV were analyzed in 10 individual DMSO- or Jas-treated cells. Each experiment was performed in triplicate and the results were presented as the mean ± SD. **(D–G)** FMDV infection was inhibited by Jas. **(D–F)** Pretreated cells (Jas) were infected (MOI 1) for 4 h at 37 °C and analyzed by RT-PCR (**D**), Western blot (**E**), and TCID50 assay (**F**). **(G)** Pretreated cells (0.2 μM Jas) were infected (MOI 25) for 4 h at 37 °C and processed for confocal microscopy as in (**B**). **(H)** Effect of Jas on virus entry and post-entry steps. Cells were treated with Jas 30 min before the infection (Pre) or treated 60 min after virus addition (Post) and maintained during the infection. Cells were then infected (MOI 1) for 4 h at 37 °C and processed for Western blot analysis. 3D, FMDV 3D; β-actin, load control; SD, standard deviation; *P < 0.05; **P < 0.01.

To further prove that FMDV internalization can induce plasma membrane ruffling, BHK-21 cells were infected with FMDV (MOI 100). After adsorption at 4 °C for 60 min, the cells were shifted to 37 °C for 5 min. The cells were fixed and observed by TEM. Viral particles were adsorbed near the ruffles (Fig. 4A). These characteristics were highly similar to those of macropinocytosis.

Macropinocytosis is heavily dependent on actin. Actin is a component of membrane ruffles, and actin rearrangement is necessary for membrane ruffling formation^13^,^14^. To determine whether FMDV depends on actin to enter cells, we used jasplakinoline (Jas), which disrupts actin filaments and induces polymerization of monomeric actin into amorphous masses^38^, thus preventing actin rearrangements. After BHK-21 cells were pretreated by Jas for 30 min (Supplementary Fig. 2), the cells were infected with FMDV in the presence of Jas. After 1 hpi, the cells were fixed and prepared for CLSM. Compared with DMSO-treated cells, the amount of FMDV in the Jas-treated cells was significantly reduced, with massive virus particles concentrated at the margin of the cells (Fig. 4B,C). The intracytoplasmic actin in the Jas-treated cells was also obviously disordered (Fig. 4B). These results indicated that FMDV entry into cells was mediated by actin dynamics. RT-PCR, Western blot, and TCID50 assays were also performed after 4 hpi. These results showed that Jas treatment could significantly inhibit FMDV infection in a dose-dependent manner (Fig. 4D–F), and CLSM also yielded consistent results (Fig. 4G). Jas treatment at 60 min after FMDV addition barely induced any changes in the amount of FMDV (Fig. 4H). Similar results were also observed with Jas-treated PK cells (Supplementary Fig. 3D). These findings indicated that Jas only interfered with FMDV entry but not the post-entry steps. Therefore, actin dynamics was involved in FMDV entry into cells.

### FMDV entry depends on NHE and stimulates fluid-phase uptake

**Figure 5:**
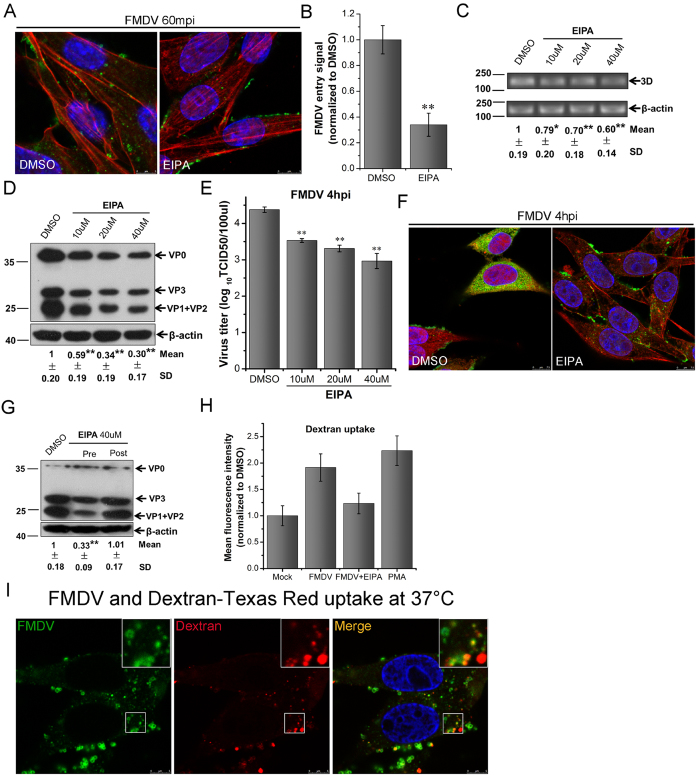
EIPA inhibits FMDV entry into BHK-21 cells and FMDV stimulates fluid-phase uptake. **(A)** EIPA inhibited FMDV entry. Pretreated cells (40 μM EIPA) were infected (MOI 25) for 1 h at 37 °C and then processed for confocal microscopy with AF594-phalloidin (red), anti-FMDV (green), and DAPI (blue). **(B)** Quantitative analysis of the internalization of FMDV. The internalized FMDV were analyzed in 10 individual DMSO- or EIPA-treated cells. Each experiment was performed in triplicate and the results were presented as the mean ± SD. **(C–E)** Pretreated cells (EIPA) were infected (MOI 1) for 4 h at 37 °C and analyzed by RT-PCR (**C**), Western blot (**D**), and TCID50 assay (**E**). **(F)** Pretreated cells (0.2 μM EIPA) were infected (MOI 25) for 4 h at 37 °C and processed for confocal microscopy as in (**A**). **(G)** Effect of EIPA on virus entry and post-entry steps. Cells were treated with EIPA 30 min before the infection (Pre) or treated 60 min after virus addition (Post) and maintained during the infection. Cells were then infected (MOI 1) for 4 h at 37 °C and processed for Western blot analysis. **(H)** FMDV stimulated fluid-phase uptake. Cells were pretreated (40 μM EIPA) and infected (MOI 10) or stimulated with PMA for 30 min, pulsed with AF594-dextran for 15 min, and analyzed by FACS. **(I)** FMDV colocalized with dextran. FMDV (MOI 25) was allowed to bind to cells for 1 h at 4 °C. The inoculum was replaced with medium containing AF594-dextran and incubated for 15 min in 37 °C. Cells were fixed and incubated with anti-FMDV antibody (green). 3D, FMDV 3D; β-actin, load control; SD, standard deviation; *P < 0.05; **P < 0.01.

Macropinosome formation is dependent on NHE. Thus, 5-ethylisopropyl amiloride (EIPA), a specific inhibitor of NHE, is widely used to detect the occurrence of macropinocytosis^16^,^39^. To verify whether FMDV entry involves macropinocytosis, FMDV was added to BHK-21 cells previously treated with DMSO or EIPA of indicated concentrations. After 1 hpi, the cells were fixed and incubated with anti-FMDV antibody to stain the virus. Based on the results of CLSM analysis, FMDV entry was significantly reduced in EIPA-treated cells compared with that in DMSO-treated cells (Fig. 5A,B). Thus, FMDV entry required NHE. After 4 hpi, RT-PCR, Western blot, and TCID50 assay were performed. EIPA treatment significantly reduced the amount of FMDV in a dose-dependent manner (Fig. 5C–E). CLSM also revealed that viral factories clearly diminished in EIPA-treated cells (Fig. 5F). However, EIPA treatment following FMDV infection for 1 h barely caused any changes to viral production (Fig. 5G). The effects of EIPA on PK-15 cells were also similar to those on BHK-21 cells (Supplementary Fig. 3E). All these results demonstrated that FMDV entry involved NHEs.

Activation of macropinocytosis can also induce transient elevation of nonspecific fluid uptake^14^,^40^. To observe whether FMDV can induce this phenomenon, the cells were incubated with FMDV and the fluid-phase marker Alexa Fluor 594-dextran, as described in the Materials and Methods, and PMA was administered as a positive control. FACS analysis showed that FMDV infection increased dextran uptake, and this action was inhibited by EIPA treatment (Fig. 5H). CLSM also discovered the intracytoplasmic colocalization of FMDV and dextran during FMDV entry (Fig. 5I). This finding further proved that FMDV infection promoted fluid-phase uptake. Therefore, FMDV entry induced the activation of macropinocytosis.

### FMDV entry depends on RTKs

**Figure 6:**
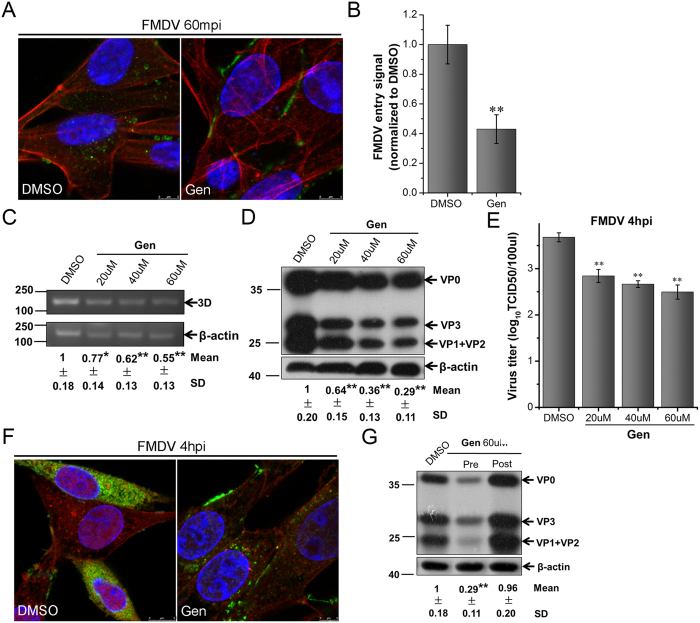
RTKs are required for FMDV entry into BHK-21 cells. **(A,B)** Gen inhibited FMDV entry. Pretreated cells (60 μM Gen) were infected (MOI 25) for 1 h at 37 °C and processed for confocal microscopy with AF594-phalloidin (red), anti-FMDV (green), and DAPI (blue). **(B)** Quantitative analysis of the internalization of FMDV. The internalized FMDV were analyzed in 10 individual DMSO- or Gen-treated cells. Each experiment was performed in triplicate and the results were presented as the mean ± SD. (**C–F)** FMDV infection was inhibited by Gen. **(C–E)** Pretreated cells (Gen) were infected (MOI 1) for 4 h at 37 °C and analyzed by RT-PCR (**C**), Western blot (**D**), and TCID50 assay (**E**). **(F)** Pretreated cells (60 μM Gen) were infected (MOI 25) for 4 h at 37 °C and processed for confocal microscopy as in (**A**). **(G)** Effect of Gen on virus entry and post-entry steps. Cells were treated with Gen 30 min before the infection (Pre) or treated 60 min after virus addition (Post) and maintained during the infection. Cells were then infected (MOI 1) for 4 h at 37 °C and processed for Western blot analysis. 3D, FMDV 3D; β-actin, load control; SD, standard deviation; *P < 0.05; **P < 0.01.

Macropinocytosis is usually initiated by external stimuli, such as growth factors or viral entry. These stimulating factors can activate RTKs (such as epithelial growth factor receptor) or integrins. Consequently, these RTKs or integrins will trigger a series of signaling cascades that lead to the formation of membrane ruffles and macropinosomes^14^,^15^,^16^. To assess the role of RTKs on FMDV entry, the cells were pretreated with broad-spectrum RTK inhibitor genistein (Gen) and then inoculated with FMDV, as described in the Materials and Methods. Cell vitality of treated cells was not significantly affected by 40 μM Gen (Supplementary Fig. 2). At 1 h after FMDV infection, CLSM revealed that Gen treatment significantly inhibited FMDV entry, thereby suggesting the importance of RTK activation (Fig. 6A,B). After 4 hpi, the amount of FMDV in cells was detected. Compared with DMSO, Gen significantly reduced viral production in a dose-dependent manner (Fig. 6C–F). To determine the specific stage of FMDV infection at which RTK participates, Gen treatment was performed 30 min before FMDV addition or 60 min after FMDV addition. Western blot assay showed that Gen mainly influenced FMDV entry but exhibited nearly no effect on FMDV post-entry (Fig. 6G). By contrast, Gen treatment barely influenced FMDV entry in PK-15 cells (Supplementary Fig. 3F). These results suggested that FMDV entry depended on RTK activation but in a cell type-dependent manner.

### PI3K is not required for FMDV entry

**Figure 7:**
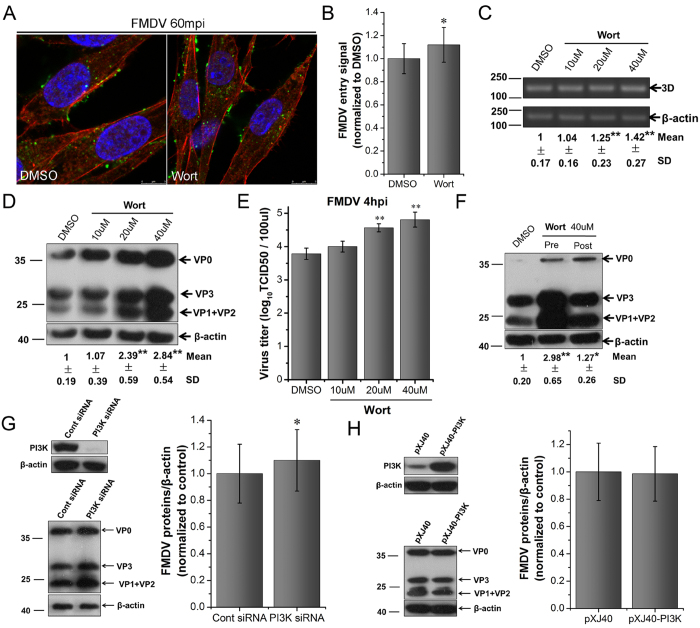
PI3K is not required for FMDV entry and replication in BHK-21 cells. **(A,B)** Wort moderately stimulated FMDV entry. Pretreated cells (40 μM Wort) were infected (MOI 25) for 1 h at 37 °C and processed for confocal microscopy with AF594-phalloidin (red), anti-FMDV (green), and DAPI (blue). **(B)** Quantitative analysis of the internalization of FMDV. The internalized FMDV were analyzed in 10 individual DMSO- or Wort-treated cells. Each experiment was performed in triplicate and the results were presented as the mean ± SD. (**C–E)** Wort enhanced FMDV infection. Pretreated cells (Wort) were infected (MOI 1) for 4 h at 37 °C and analyzed by RT-PCR (**C**), Western blot (**D**), and TCID50 assay (**E**). **(F)** Effect of Wort on virus entry and post-entry steps. Cells were treated with Wort 30 min before the infection (Pre) or treated 60 min after virus addition (Post) and maintained during the infection. Cells were then infected (MOI 1) for 4 h at 37 °C and processed for Western blot analysis. **(G)** PI3K downregulation moderately enhanced FMDV infection. **(H)** PI3K overexpression did not affect FMDV infection. 3D, FMDV 3D; β-actin, load control; SD, standard deviation; *P < 0.05; **P < 0.01.

PI3K is involved in multiple stages of macropinocytosis, including the formation of membrane ruffles, as well as closure, trafficking, and fusion of macropinosomes^16^. Recent studies reported that PI3K plays a vital role in macropinocytosis of vaccinia virus^13^, African swine fever virus (ASFV)^41^, and respiratory syncytial virus (RSV)^42^. Whether FMDV internalization involves PI3K was determined by treating the cells with specific PI3K inhibitor wortmannin (Wort) at low concentration to prevent cytotoxicity (Supplementary Fig. 2). Wort-treated BHK-21 cells were infected with FMDV. After 1 hpi, CLSM analysis revealed that Wort treatment moderately promoted FMDV internalization to a certain extent (Fig. 7A,B). After 4 hpi, Wort treatment did not inhibit FMDV infection, as shown by RT-PCR, Western blot, and TCID50 assays. By contrast, a high concentration of Wort promoted FMDV infection (Fig. 7C–E). To further elucidate the time point when Wort affects FMDV infection, the cells were treated with Wort before and after FMDV addition as previously described. Wort mainly influenced the entry step of FMDV infection (Fig. 7F). Consistent with Wort-treated BHK-21 cells, Wort-treated PK-15 cells also presented an increase in viral infection (Supplementary Fig. 3G). To further determine the role of PI3K in FMDV infection, we down-regulated or up-regulated the expression levels of PI3K with specific siPI3K and plasmid pXJ40-PI3K, respectively. Compared with the control siRNA, PI3K siRNA moderately enhanced viral production (Fig. 7G). However, up-regulation of PI3K expression did not influence FMDV infection (Fig. 7H). Overall, these results indicated that FMDV entry did not involve PI3K, and the inhibition of PI3K activity could moderately promote FMDV internalization.

### FMDV entry activates Rac1 and depends on Dynamin II

**Figure 8:**
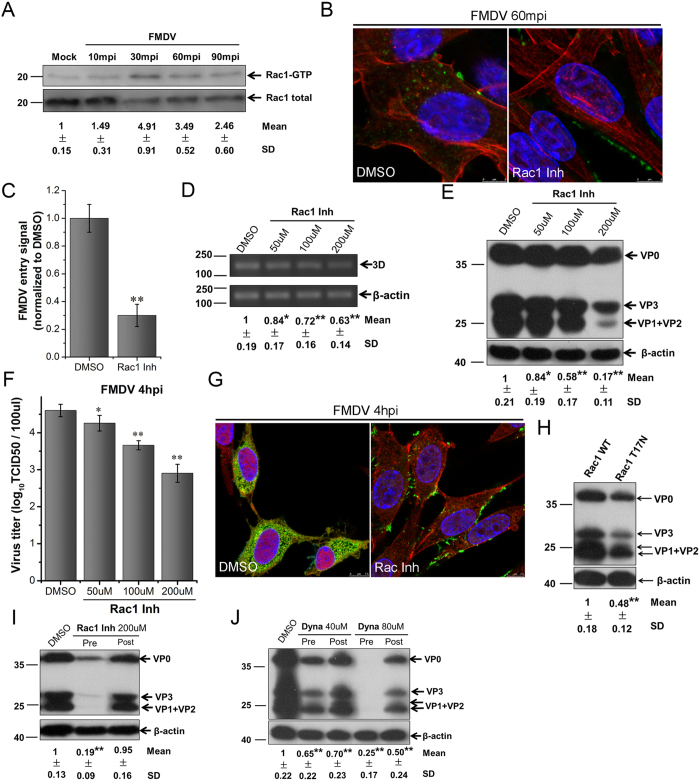
FMDV entry into BHK-21 cells activates Rac1 and depends on Dynamin II. **(A)** Activation of Rac1 during FMDV entry. Cells were infected (MOI 10), and Rac1 activation was measured by GST-PAK1-PBD pull-down assay. Fold induction was determined by densitometry. **(B,C)** Rac1 Inh inhibited FMDV entry. Pretreated cells (200 μM Rac1 Inh) were infected (MOI 25) for 1 h at 37 °C and processed for confocal microscopy with AF594-phalloidin (red), anti-FMDV (green), and DAPI (blue). **(C)** Quantitative analysis of the internalization of FMDV. The internalized FMDV were analyzed in 10 individual DMSO- or Rac1 Inh-treated cells. Each experiment was performed in triplicate and the results were presented as the mean ± SD. **(D–G)** FMDV infection was inhibited by Rac1 Inh. **(D–F)** Pretreated cells (Rac1 Inh) were infected (MOI 1) for 4 h at 37 °C and analyzed by RT-PCR (**D**), Western blot (**E**), and TCID50 assay (**F**). **(G)** Pretreated cells (200 μM Rac1 Inh) were infected (MOI 25) for 4 h at 37 °C and processed for confocal microscopy as in (**B**). **(H)** Expression of inactive form of Rac1 inhibited FMDV infection. Transfected cells with Rac1 (WT) and Rac1 (T17N) were infected (MOI 1) for 4 h at 37 °C and analyzed by Western blot. **(I)** Effect of Rac1 Inh on virus entry and post-entry steps. Cells were treated with Rac1 Inh 30 min before the infection (Pre) or treated 60 min after virus addition (Post) and maintained during the infection. Cells were then infected (MOI 1) for 4 h at 37 °C and processed for Western blot analysis. **(J)** Dynasore (Dyna) inhibited FMDV entry and multiplication. Cells were treated with indicated concentrations of Dyna as in (I) and then processed for Western blot analysis. 3D, FMDV 3D; β-actin, load control; SD, standard deviation; *P < 0.05; **P < 0.01.

Once activated, Rac1, as a Rho GTPase, can regulate the polymerization of actins on the cell membrane, as well as the contractibility of myosin. In this way, the formation of membrane ruffles and closure of macropinosomes are induced^15^,^43^,^44^. In the present experiment, the activation status of Rac1 at the first step of FMDV entry into BHK-21 cells was investigated. Rac1-GTP in activated form can bind to and activate the Pak1. Thus, the GST-Pak1-PBD beads were applied for pull-down test to detect the activation level of Rac1 at indicated time points of early FMDV entry. The activation level of Rac1 slightly increased at 10 min after FMDV entry compared with the control (Fig. 8A). At 30 mpi, the activation level reached the peak (about five times that of the control). FMDV infection induced Rac1 activation, which occurred mainly during FMDV entry.

The role of Rac1 in FMDV infection was also investigated. BHK-21 cells were treated with Rac1 inhibitor (Rac1 Inh) and infected with FMDV. At 1 h of FMDV infection, CLSM showed that the amount of FMDV obviously decreased in the presence of Rac1 inhibitor (Supplementary Fig. 2), and a large number of FMDVs adhered to the cell membrane (Fig. 8B,C). At 4 h after FMDV infection, CLSM showed no viral factories in cells treated with Rac1 inhibitor (Fig. 8G). These findings were consistent with the results of RT-PCR, Western blot, and TCID50 assays (Fig. 8D–F). To reinforce the role of Rac1 in FMDV infection, the cells were transfected using the plasmids pCyPet-Rac1 (WT) and pCyPet-Rac1 (T17N) for 30 h and then infected with FMDV for 4 h. The data (Fig. 8H) showed that the inactive form of Rac1 (T17N) significantly inhibited FMDV infection. When Rac1 inhibitor was added 60 min after viral addition, the synthesis of viral proteins was unaffected (Fig. 8I). Similar results were obtained from the PK-15 cells treated with Rac1 inhibitor (Supplementary Fig. 3H). Overall, Rac1 activation was a crucial step in viral entry.

Dynamin II is involved in the regulation of Rac1 localization and functions^45^. To verify the effects of dynamin II on FMDV infection, dynasore (Dyna), a reversible inhibitor of dynamin II^46^, was used to treat BHK-21 cells at indicated time periods. At 4 h after infection, Western blot assay was performed to detect the expression levels of FMDV proteins. Dynasore treatment before or after FMDV addition significantly blocked FMDV infection in BHK-21 cells in a dose-dependent manner (Fig. 8J). However, dynasore exhibited a weaker inhibitory effect on FMDV infection in PK-15 cells than that in BHK-21 cells (Supplementary Fig. 3I). Dynasore at 80 μM also slightly influenced the vitality of BHK-21 and PK-15 cells (Supplementary Fig. 2). All these results demonstrated that FMDV infection depended on dynamin II in both viral entry and post-entry steps in a cell type-dependent manner.

### Pak1 activation is required for FMDV entry

**Figure 9:**
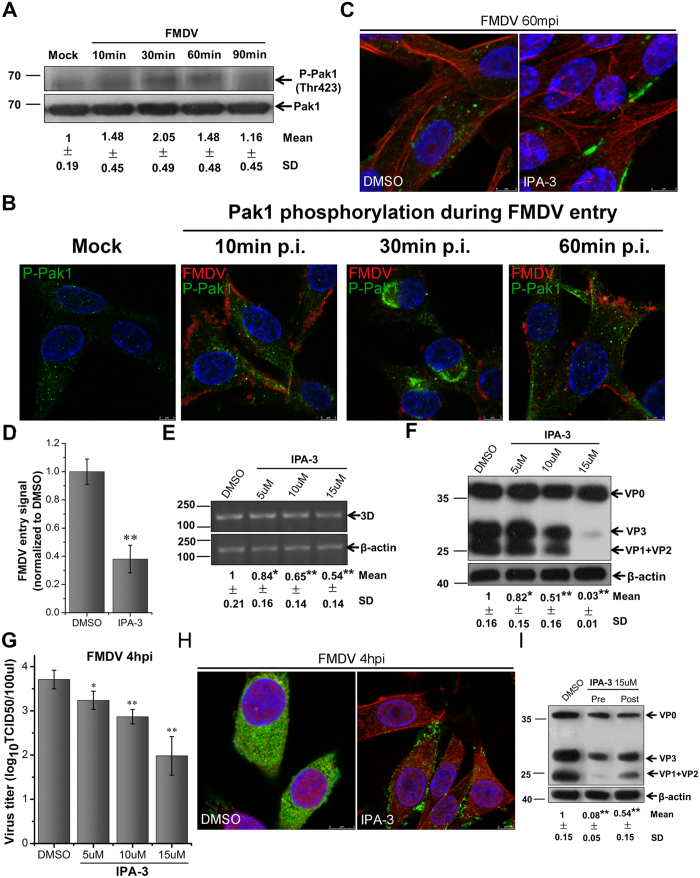
Pak1 is required for FMDV entry into BHK-21cells. **(A,B)** FMDV activated Pak1 during early post-infection. **(A)** Cells were infected (MOI 10), and phosphorylation of Pak1 (Thr423) was determined at different times after infection by Western blot analysis. The level of total Pak1 was measured as the control. Fold induction was determined by densitometry. **(B)** Cells were infected (MOI 25) and processed for confocal microscopy with anti-phospho-Pak1 (green), anti-FMDV (red), and DAPI (blue). **(C,D)** IPA-3 inhibited FMDV entry. Pretreated cells (15 μM IPA-3) were infected (MOI 25) for 1 h at 37 °C and processed for confocal microscopy with AF594-phalloidin (red), anti-FMDV (green), and DAPI (blue). **(D)** Quantitative analysis of the internalization of FMDV. The internalized FMDV were analyzed in 10 individual DMSO- or IPA-3-treated cells. Each experiment was performed in triplicate and the results were presented as the mean ± SD. (**E–H**) FMDV infection was inhibited by IPA-3. **(E–G)** Pretreated cells (IPA-3) were infected (MOI 1) for 4 h at 37 °C and analyzed by RT-PCR (E), Western blot (F), and TCID50 assay (G). **(H)** Pretreated cells (15 μM IPA-3) were infected (MOI 25) for 4 h at 37 °C and processed for confocal microscopy as in (**C**). **(I)** Effect of IPA-3 on virus entry and post-entry steps. Cells were treated with IPA-3 30 min before the infection (Pre) or treated 60 min after virus addition (Post) and maintained during the infection. Cells were then infected (MOI 1) for 4 h at 37 °C and processed for Western blot analysis. 3D, FMDV 3D; β-actin, load control; SD, standard deviation; *P < 0.05; **P < 0.01.

The P21-activated kinase 1 (Pak1) is a serine/threonine kinase activated by Rho GTPase Rac1 or Cdc42. Activated Pak1 is translocated to the cell membrane where it activates various effectors required for the regulation of cytoskeletal dynamics, ruffle formation, and macropinosome closure^47^,^48^. Phosphorylation at some residues of Pak1, specifically T423, is crucial for the full activation of Pak1^49^. To test whether FMDV infection can activate Pak1, BHK-21 cells were serum starved for 1 h and then infected with FMDV for different times. Western blot analyses showed that the phosphorylation level of Pak1 (T423) increased rapidly after FMDV infection. Reaching the peak at 30 mpi (about 2.2 times that of the control), the phosphorylation level gradually decreased until it restored to the initial state at 90 mpi (Fig. 9A). To further confirm that FMDV infection can induce Pak1 activation, the antibody against Phospho-Pak1 (P-Pak1) was used for immunofluorescence experiment. The fluorescence signals of P-Pak1 were weaker in normal cells (Fig. 9B). At 10 min after FMDV infection, the specific fluorescence signals enhanced rapidly. After 30 mpi, the fluorescence signals became brighter and were found near the nuclei. The signals were then gradually weakened until 60 mpi. This result was consistent with that of Western blot assay, which suggested the significant role of Pak1 activation in FMDV entry.

IPA-3 is the allosteric inhibitor of Pak1, which specifically binds to the autoinhibitory domain of Pak1 and inhibits the activation of Pak1^50^. To further investigate the effect of Pak1 activation on FMDV uptake, we treated BHK-21 cells with IPA-3 30 min before FMDV infection, and IPA-3 was maintained during the whole infection period. After 1 hpi, CLSM analysis showed that IPA-3 significantly inhibited FMDV internalization (Fig. 9C,D). After 4 hpi, detection of the levels of viral genes, proteins, and titers showed that FMDV production was severely inhibited by IPA-3 in a dose-dependent manner (Fig. 9E–G). Similarly, viral factories disappeared in IPA-3-treated cells (Fig. 9H). To determine whether IPA-3 influences the multiplication stage, the cells were treated with IPA-3 at 1 h after FMDV infection. Western blot analyses showed that IPA-3 decreased the amount of viral proteins by about 50% (Fig. 9I). Similar results were obtained in PK-15 cells treated with IPA-3 (Supplementary Fig. 3J), and the cytotoxic effects of IPA-3 on BHK-21 and PK-15 cells were excluded (Supplementary Fig. 2). These results indicated that FMDV activated Pak1, which consequently mediated FMDV entry and multiplication. By combining with the Rac1 activation experiment, FMDV infection could activate the Rac1-Pak1 signaling pathway.

### PKC is required for FMDV entry

**Figure 10:**
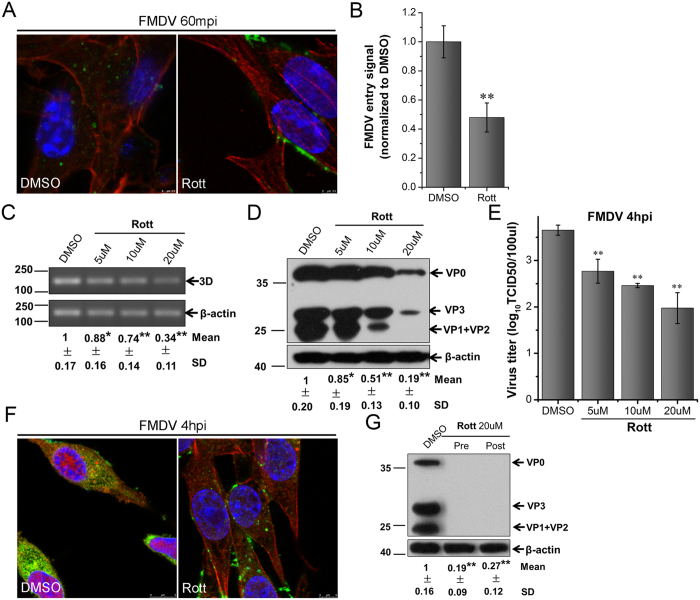
PKC is required for FMDV entry and multiplication in BHK-21 cells. **(A,B)** Rott inhibited FMDV entry. Pretreated cells (20 μM Rott) were infected (MOI 25) for 1 h at 37 °C and processed for confocal microscopy with AF594-phalloidin (red), anti-FMDV (green), and DAPI (blue). **(B)** Quantitative analysis of the internalization of FMDV. The internalized FMDV were analyzed in 10 individual DMSO- or Rott-treated cells. Each experiment was performed in triplicate and the results were presented as the mean ± SD. (**C–F)** FMDV infection was inhibited by Rott. **(C–E)** Pretreated cells (Rott) were infected (MOI 1) for 4 h at 37 °C and analyzed by RT-PCR (**C**), Western blot (**D**), and TCID50 assay (**E**). **(F)** Pretreated cells (20 μM Rott) were infected (MOI 25) for 4 h at 37 °C and processed for confocal microscopy as in (**A**). **(G)** Effect of Rott on virus entry and post-entry steps. Cells were treated with Rott 30 min before the infection (Pre) or treated 60 min after virus addition (Post) and maintained during the infection. Cells were then infected (MOI 1) for 4 h at 37 °C and processed for Western blot analysis. 3D, FMDV 3D; β-actin, load control; SD, standard deviation; *P < 0.05; **P < 0.01.

PKC is a Ca2^+^- and diacylglycerol-dependent serine/threonine kinase, the activation of which can promote the formation of membrane ruffles and macropinosomes^51^. Thus, the PKC-specific inhibitor rottlerin (Rott) was employed and its effect on FMDV infection was observed. The BHK-21 cells were treated with 20 μM Rott and infected with FMDV. At the indicated time points, CLSM revealed that Rott inhibited FMDV internalization (Fig. 10A,B), and viral factories disappeared in Rott-treated cells (Fig. 10F). Furthermore, the results of RT-PCR, Western blot and TCID50 assay showed that Rott strongly decreased viral production in a dose-dependent manner (Fig. 10C–E). The administration of Rott after FMDV addition decreased the amount of viral proteins by approximately 70% (Fig. 10G). Similar results were observed in PK-15 cells treated with Rott (Supplementary Fig. 3K), and the cytotoxic effect of Rott on BHK-21 and PK-15 cells was excluded (Supplementary Fig. 2). These results implied that PKC was involved in FMDV entry and played a critical role in FMDV multiplication.

### FMDV entry depends on myosin II

**Figure 11:**
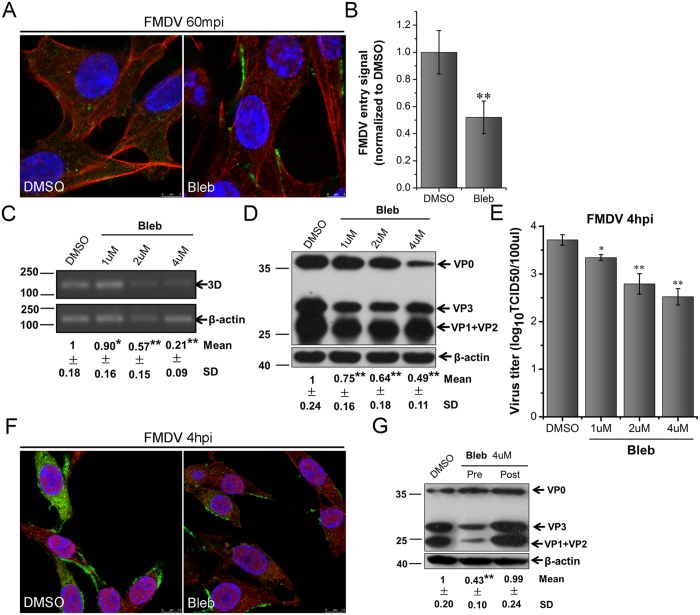
Myosin II is required for FMDV entry in BHK-21 cells. **(A,B)** Bleb inhibited FMDV entry. Pretreated cells (4 μM Bleb) were infected (MOI 25) for 1 h at 37 °C and processed for confocal microscopy with AF594-phalloidin (red), anti-FMDV (green), and DAPI (blue). **(B)** Quantitative analysis of the internalization of FMDV. The internalized FMDV were analyzed in 10 individual DMSO- or Bleb-treated cells. Each experiment was performed in triplicate and the results were presented as the mean ± SD. (**C–F)** FMDV infection was inhibited by Bleb. **(C–E)** Pretreated cells (Bleb) were infected (MOI 1) for 4 h at 37 °C and analyzed by RT-PCR (C), Western blot (D), and TCID50 assay (E). **(F)** Pretreated cells (4 μM Bleb) were infected (MOI 25) for 4 h at 37 °C and processed for confocal microscopy as in (**A**). **(G)** Effect of Bleb on virus entry and post-entry steps. Cells were treated with Bleb 30 min before the infection (Pre) or treated 60 min after virus addition (Post) and maintained during the infection. Cells were then infected (MOI 1) for 4 h at 37 °C and processed for Western blot analysis. 3D, FMDV 3D; β-actin, load control; SD, standard deviation; *P < 0.05; **P < 0.01.

Myosins are present in membrane ruffles. Modulated by Pak1, they provide contractile activity that can regulate the movement of ruffles and closure of macropinosomes^15^,^52^,^53^. To test whether FMDV entry into BHK-21 cells also depends on myosin II, the effect of blebbistatin (Bleb), a cell-permeable nonmuscle myosin II inhibitor, was investigated. First, we analyzed changes in FMDV uptake in the presence of Bleb by CLSM. As Fig. 11A indicated, Bleb treatment could induce the strong inhibition of FMDV uptake, because the number of virus particles in the Bleb-treated cells was visibly lower than that of the DMSO-treated cells (Fig. 11A,B). Moreover, the results of RT-PCR, Western blot, and TCID50 assays proved that Bleb significantly reduced FMDV production in a dose-dependent manner (Fig. 11C–E). The viral factories also disappeared in the Bleb-treated cells (Fig. 11F). Bleb treatment at 60 min after FMDV addition barely induced any changes in viral protein expression (Fig. 11G). However, Bleb treatment had no effect on the occurrence of FMDV infection in PK-15 cells (Supplementary Fig. 3L). Furthermore, when tested on BHK-21 and PK-15 cells, Bleb had no significant cytotoxic effects (Supplementary Fig. 2). These results indicated that FMDV entry involved myosin II via a cell type-dependent mechanism.

### Intracellular trafficking of FMDV involves EEA1/Rab5-positive vesicles

By combining the aforementioned findings, FMDV was inferred to enter host cells via macropinocytosis. However, intracellular transport required for FMDV infection remains largely unknown. Previous studies indicated that after FMDV internalization via the clathrin-mediated pathway, the virus is rapidly transported to the early endosome and subsequently to the recycling endosome, but never to the late endosome^54^,^55^,^56^. However, HS-binding FMDV moves via the caveolin-dependent pathway at a rate slower than that of the integrin-binding FMDV. The HS-binding FMDV moves via the early endosome into the recycling endosome and Golgi apparatus^31^. Given the extreme sensitivity of FMDV to acidic pH, the low pH environment in early endosomes is thought to be a prerequisite for FMDV uncoating^54^,^55^.

**Figure 12:**
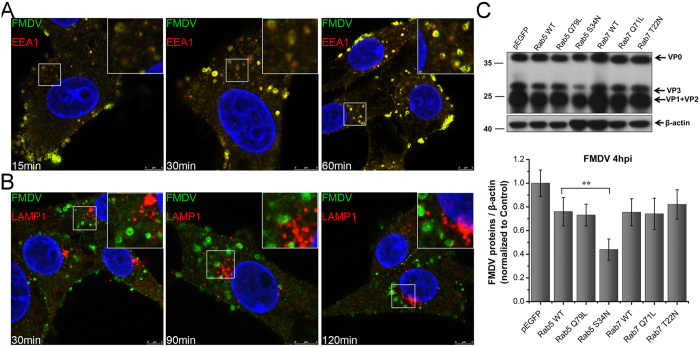
Intracellular trafficking of FMDV involves EEA1/Rab5-positive vesicles but does not involve LAMP1/Rab7-positive vesicles. **(A)** FMDV colocalized with EEA-1 positive vesicles. BHK-21 cells were infected (MOI 25) for different time intervals and processed for confocal microscopy with anti-EEA1 (red), anti FMDV (green), and DAPI (blue). **(B)** FMDV did not colocalize with LAMP-1 positive vesicles. FMDV-infected cells (MOI 25) were processed for confocal microscopy with anti-LAMP1 (red), anti FMDV (green), and DAPI (blue). **(C)** FMDV traffic required Rab5 function. BHK-21 cells were transfected to express pEGFP or pEGFP-tagged forms of Rab5 WT, Rab5 Q79L, Rab5 S34N, Rab7 WT, Rab7 Q71L, and Rab7 T22N. The plasmid-transfected cells were then infected (MOI 1) for 4 h at 37 °C and analyzed by Western blot.

The intracellular trafficking mechanism of macropinosomes is still unclear, but macropinosomes have been shown to follow a fate similar to that of endosomes in some aspects. Their maturation program undergoes acidification and conversion between the marker proteins of early and late vesicles, such as the conversions of EEA1–LAMP1 and Rab5–Rab7^57^,^58^. To characterize the intracellular transport of FMDV in macropinosomes, BHK-21 cells were infected with FMDV. At the indicated time points, cells were fixed and labeled with an anti-FMDV antibody and antibodies for the early vesicle marker EEA1 or late vesicle marker LAMP1. Within the 15–60 min incubation period, FMDV was almost fully colocalized with EEA1-positive vesicles (Fig. 12A). However, during the complete incubation process, FMDV rarely colocalized with the LAMP1-positive vesicles (Fig. 12B).

In addition, multiple transfection reactions were prepared to investigate the role of Rab5 and Rab7 in FMDV infection. The results in Fig. 12C showed that constitutively active (CA) GFP–Rab5 (Q79L), DN GFP–Rab7 (T22N), and CA GFP–Rab7 (Q71L) barely influenced FMDV infection. However, DN GFP–Rab5 (S34N) significantly inhibited FMDV infection. These results agreed with those of Johns *et al.*^56^. Furthermore, the EEA1-positive compartment and Rab5 were necessary during the intracellular trafficking process of FMDV. However, the LAMP1-positive compartment and Rab7 were not necessary. Therefore, the same trafficking proteins were probably involved in the intracellular transport of macropinosomes and endosomes.

To further investigate the effect of intracellular vesicle trafficking on FMDV infection, the cells were treated with nocodazole (Noc), which is a microtubule-depolymerizing agent that can block intracellular vesicle trafficking and cause the retention of cargos, such as viruses, in early vesicles. CLSM revealed that Noc destroyed the microtubule network of cells. However, such destruction had no obvious effects on FMDV infection (Supplementary Fig. 4). Furthermore, FMDV did not enter the late macropinosomes labeled by LAMP1/Rab7. FMDV possibly already finished its uncoating during the early maturity stage of macropinosomes.

## Discussion

Most animal viruses take advantage of the endocytotic activities of a cell for entry and infection. Endocytic processes provide several advantages to incoming viruses, including the ability to pass through the plasma membrane barrier and underlying cortical matrix. The viral genome can then be transported to the preferred subcellular sites to start viral replication. An investigation of the viral invasion pathway and exact regulatory mechanism is necessary for understanding the viral pathogenic mechanism. Moreover, these findings can be used to screen novel targets to prevent the virus from commandeering the host cell machinery for replication.

Early studies showed that the field isolates of FMDV invade the cells via a CME pathway involving the integrin receptor^27^,^28^,^29^,^30^. Cell culture-adapted isolates bind to the HS receptor and enter the cell via the caveolin-mediated endocytic pathway^31^. However, some FMDV isolates can still preserve their infectivity to host cells even after the clathrin- and caveolin-mediated endocytic pathways are blocked^32^,^59^,^60^. Consequently, we proposed the possible existence of alternative endocytic pathways for FMDV. Previous reports demonstrated that some picornaviruses, such as EV1, CVB and CVA9, utilize macropinocytosis to invade cells^23^,^24^,^61^,^62^. The possibility that FMDV also utilizes macropinocytosis to invade cells was tested. To prove this hypothesis, different and independent approaches were combined to obtain an exhaustive analysis of the endocytic pathway of FMDV.

The present results indicated that FMDV could enter cells via the clathrin-mediated pathway but not the caveolin-mediated endocytic pathway. However, the former was not the only endocytic pathway of FMDV. The specific blocking of the CME pathway reduced FMDV production by approximately 30%. During the internalization process, only a small portion of FMDV was colocalized with TF. In addition, the specific blocking of the caveolin-mediated endocytic pathway did not affect FMDV infection. During the invasion process, FMDV was not colocalized with CTxB. Besides, FMDV entry presented some important features of macropinocytosis. During the early stage of FMDV entry, actin rearrangement occurred, with the appearance of a large number of membrane ruffles. The dynamics of the specific inhibition of actin significantly reduced the internalization and infection of FMDV. FMDV entry caused a transient increase in fluid-phase uptake, and the internalized viral particles were colocalized with dextran. EIPA is the main diagnostic agent of macropinocytosis, which significantly inhibits FMDV and dextran uptake. After the activation of RTKs, a multi-branched signaling cascade occurred, which involved Rac1, Pak1, PKC, dynamin-2, and myosin II. These signaling proteins are responsible for the regulation of actin rearrangement, macropinosome closure, and macropinosome trafficking. The specific inhibition of these signaling factors could greatly inhibit the endocytosis and infection of FMDV. All these observations conformed to the diagnostic criteria of macropinocytosis^15^,^16^.

**Figure 13:**
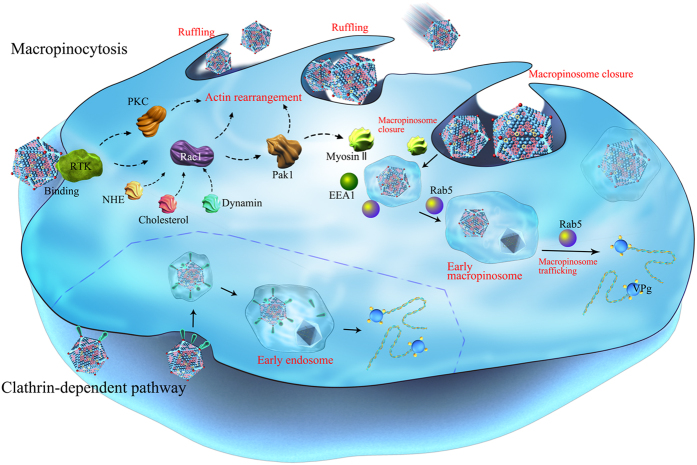
Model of the FMDV internalization pathway. FMDV entered host cells by macropinocytosis and CME. For the macropinocytic entry, the binding of FMDV to RTKs may activate cellular actin modulators (Rac1, Pak1, and PKC) and other factors, such as NHEs and dynamin, which trigger actin rearrangement and plasma membrane ruffling. The virions are then internalized into macropinosomes, and the membrane fission events that separate the macropinosomes from the extracellular space occur in a myosin II-dependent manner. After closure, the early macropinosomes containing FMDV acquire Rab5 and EEA1, which facilitate intracellular trafficking. The acidic pH of macropinosomes may trigger viral uncoating. As an alternative entry route of FMDV, virion binding to integrin receptors induces viral internalization via CME. The internalized vesicle is then delivered to early endosomes, and the endosomal acidic pH triggers viral uncoating.

By combining all our findings in this report, we have generated a model of FMDV entry into the cell and identified the main required cellular proteins (Fig. 13), which are described in more detail below.

Similar to other viruses, FMDV can utilize two endocytic pathways to enter cells. Our results indicated that aside from the clathrin-mediated pathway, FMDV could utilize macropinocytosis. During FMDV infection, dynamin and membrane cholesterol are highly important. Dynamin GTPase mediates the dissociation of the newly formed endocytic vesicles from the cell membrane, which is a necessary step in the clathrin- and caveolin-mediated endocytic pathways^63^. Dynamin II is involved in the regulation of the localization and functions of Rac1^45^. Activated Rac1 is an important signaling factor in the regulation of macropinocytosis. These events account for the role of dynamin II in FMDV infection to a certain extent. Membrane cholesterol plays important roles in various endocytic pathways, including the caveolin-mediated endocytic and CME pathways and macropinocytosis^8^. The reorganization of the membrane lipid raft did not affect FMDV entry and progression. Therefore, FMDV did not depend on caveolin-mediated endocytosis to enter cells. However, the depletion of cholesterol seriously inhibited viral internalization and multiplication. The depletion of cholesterol probably caused phosphoinositide rearrangement in the cell membrane, which affected the localization of signaling molecules, such as Rac1^64^. Consequently, the formation of membrane ruffles and macropinosomes was prevented. In addition, the formation of clathrin-coated vesicles might also involve cholesterol^65^.

Macropinocytosis is also a receptor-mediated endocytic pathway. Once activated by viruses, macropinocytosis involves the engagement of various cell-surface receptors, including the RTKs, integrin, or PS receptors^14^,^15^,^16^. As the largest class of enzyme-linked receptors, RTKs are common receptors used to initiate a multi-branched signaling cascade of macropinocytosis. Several viruses have been found to recruit RTKs to achieve productive entry, including the influenza A viruses^66^, ASFV^41^, and RSV^42^. Our present results indicated that RTKs were required for FMDV entry, with cellular specificity. However, the RTK family member that participates in FMDV entry remains unknown. Integrin is a known receptor of FMDV, but its involvement in viral macropinocytosis and the relationship between integrin and RTKs during virus entry require further study.

The activation of macropinocytosis via the virus-induced engagement of RTKs initiates several parallel signaling cascades that involve PI3K and Ras-GTPase Rac1, which are known important signaling molecules that regulate macropinocytosis^15^,^16^. Recent reports suggested that viruses may utilize the PI3K-AKT pathway when entering cells via macropinocytosis^13^,^41^,^42^. However, macropinocytosis can occur in the presence of the PI3K inhibitor Wort in viruses, such as the vaccinia virus IHD-J EVs, MVs^18^, and CVA9^62^. Other studies proposed that FMDV enters cells and induces autophagosomes via a PI3K-independent pathway^67^,^68^. Our present results showed that PI3K was not only irrelevant with FMDV entry, but viral infection was promoted by blocking PI3K activity. These results implied that other lipid kinases may regulate macropinocytosis. By inhibiting PI3K activity, these kinase-induced signaling pathways were probably initiated to promote virus macropinocytosis in a compensatory manner. The inhibited PI3K activity may have promoted the binding of FMDV 2C with beclin-1, which further inhibited the fusion of lysosomes and autophagosomes, thereby contributing to viral survival and multiplication^69^.

Recent reports indicated that Rac1 is activated during the macropinocytosis of several viruses, such as EV1^61^, vaccinia virus^13^, ASFV^41^, and Ebola virus^19^. By interacting with its specific effector Pak1, the active Rac1 regulates the polymerization and reconstruction of the actin cytoskeleton on the plasma membrane, which is responsible for the formation of lamellipodia and circular ruffles^15^,^43^,^44^. Moreover, the active Rac1 is also a component of membrane ruffles^13^. Our results showed that FMDV caused the rapid activation of Rac1 upon entry, and the peak was reached at approximately 30 min. However, FMDV infection was greatly inhibited in cells transfected with DN-Rac1 (T17N) or treated with the Rac1 inhibitor. Therefore, Rac1 played a crucial role in productive FMDV entry. As a downstream target protein of Rac1, Pak1 is involved in actin cytoskeleton dynamics and all phases of macropinocytosis^47^,^48^. A previous study showed that EV1-infected cells induced a rapid nuclear localization of Pak1 and increased the level of phospho-Pak1 in cells, particularly in the periphery of the cells^61^. Similarly, FMDV-infected cells caused the rapid phosphorylation of Pak1, while with the active P-Pak1 concentrated near the nuclei. The Pak1 inhibitor IPA-3 significantly inhibited the entry of FMDV and its subsequent multiplication. These results further indicated that Pak1 was one of the important kinases that regulate the macropinocytic pathway of FMDV. Moreover, the Pak1-modulated active myosin in virus-induced membrane ruffles could provide contractile activity for ruffle curvature formation and macropinosome closure^15^. However, whether the FMDV entry into the cells involves myosin II remains to be further studied. As shown by our present results, the effect of myosin II on FMDV entry presented cellular specificity. To conclude, FMDV entry required activation of the Rac1-Pak1 signaling pathway.

Actin plays an important role in the formation and trafficking of macropinosomes, and actin rearrangement is a prerequisite for the occurrence of macropinocytosis^15^,^16^. At 15 min after FMDV entry into BHK-21 cells, actin rearrangement was triggered, with the formation of a large number of membrane ruffles. TEM images confirmed that FMDV was engulfed by the cells via the contraction of membrane ruffles. When the depolymerization of actin microfilaments was prevented by Jas, the viral infection was strongly inhibited. Therefore, the dynamic changes in actin promoted FMDV entry.

Macropinocytosis is the only endocytic pathway sensitive to the deactivation of NHE. EIPA is frequently used as the main indicator of virus macropinocytosis. A recent report showed that EIPA can block the activation of the Rac1 and Cdc42 signaling pathways by lowering submembranous pH, thereby inhibiting the occurrence of macropinocytosis^39^. In the present study, EIPA significantly inhibited FMDV entry in a dose-dependent manner. The results suggested that NHE was involved in macropinocytosis-mediated FMDV entry into cells.

After being activated by RTK or PI3K, PKC is implicated in signal transduction and amplification, thereby promoting the formation of membrane ruffles and macropinosomes^51^. Our results showed that PKC deactivation had an obvious effect on FMDV entry and multiplication. Similar to other viruses, the phosphorylation of PKC was involved in the entire replication cycle of FMDV. However, its specific role needs further clarification.

The critical final event in FDMV entry is uncoating, which is the release of the infectious RNA genome from the capsid. After the FMDV-containing macropinosomes were dissociated from the plasma membrane, they were soon colocalized with EEA1-labeled early vesicles but did not colocalize with LAMP1-positive late vesicles during the whole infection period. This phenomenon may be attributed to two reasons. After their formation, macropinosomes possibly acquired the EEA1-labelled protein, or they may have rapidly fused with the EEA1-labeled vesicles. The overexpression of the DN Rab5 mutant inhibited FMDV infection, which indicated that FMDV had to pass through the Rab5-containing early macropinosomes to finish the productive entry. However, the mutation of DN Rab7 and Noc-induced inhibition of vesicle maturity had no effect on viral infection. Therefore, FMDV penetration may not involve macropinosome maturation beyond the Rab5-positive stages, and virus uncoating could occur in the early vesicles. This trend agreed with previous reports that early macropinosomes undergo acidification^15^ and FMDV is extremely sensitive to acid^70^.

In conclusion, the present article is a systematic study of the endocytic pathways of FMDV entry into host cells. We have demonstrated for the first time that FMDV could utilize macropinocytosis to enter cells. The relevant features and mechanisms are proposed in this paper. Although this work is only a preliminary study, the evidence of FMDV macropinocytosis provides additional insights into the mechanism of viral productive entry. Moreover, the components of host cells that are involved in this pathway may serve as the potential targets for penetration intervention. This work may be used as a reference for the prevention and control of FMDV and other picornaviruses.

## Materials and Methods

### Cells and viruses

BHK-21 and PK-15 cells were cultured in DMEM (Invitrogen) supplemented with 10% fetal bovine serum (FBS, Sigma), 100 U/ml penicillin, and 100 μg/ml streptomycin. Cells were maintained in a 5% CO_2_ incubator at 37 °C. FMDV isolate Asia1/Jiangsu/China/2005 (GenBank Accession No. EF149009) was preserved by the OIE/National Foot-and-Mouth Disease Reference Laboratory. FMDV was propagated on BHK-21 cells, and viral purification was performed by sucrose gradient centrifugation as previously described^71^. The FMDV titer was measured by 50% tissue culture infective dose (TCID50) assay.

### Inhibitors, antibodies, plasmids, and siRNAs

Chlorpromazine (CPZ), methyl-β-cyclodextrin (MβCD), nystatin (Nys), dynasore Dyna, jasplakinolide (Jas), 5-ethylisopropyl amiloride (EIPA), IPA-3, nocodazole (Noc), rottlerin (Rott), blebbistatin (Bleb), genistein (Gen), and phorbol 12-myristate 13-acetate (PMA) were purchased from Sigma. Wortmannin (Wort) and Rac1 inhibitor (Rac1 Inh) were purchased from Calbiochem. In accordance with the manufacturer’s recommendations, CPZ, MβCD, and Nys were prepared in DMEM, and the remaining pharmacological inhibitors were prepared in DMSO (AMERSCO). Transferrin (TF), cholera toxin B (CTxB), and dextran conjugated to Alexa Fluor 594 were purchased from Invitrogen.

Polyclonal pig antiserum against FMDV was produced in our laboratory (Supplementary Fig. 5). Specific antibodies against Pak1, phospho-Pak1 (Thr423), caveolin-1 (Cav-1), EEA1, GAPDH, and β-actin were purchased from Santa Cruz Biotechnology. Rabbit anti-PI3K p100α mAb was purchased from Cell Signaling Technology. Goat anti-clathrin, anti-LAMP1-Cy3, and TRITC-phalloidin were purchased from Sigma. HRP-conjugated anti-mouse, goat and pig IgG produced in rabbit, HRP-conjugated anti-rabbit IgG produced in goat, FITC-conjugated anti-pig and goat IgG produced in rabbit, FITC-conjugated anti-rabbit IgG produced in goat, Texas Red^®^-conjugated anti-swine IgG produced in rabbit, and TRITC-conjugated anti-mouse IgG produced in goat were purchased from Sigma.

Pooled validated siRNAs targeting clathrin heavy-chain (CHC) (siCHC) (catalog no. sc-35066), Cav-1 (catalog no. sc-29942), PI3K p100α (catalog no. sc-39128) and non-targeting siRNA (catalog no. sc-36869) were purchased from Santa Cruz Biotechnology. Expression plasmids encoding pCyPet-Rac1 (WT) and pCyPet-Rac1 (T17N) were kindly provided by Dr. Klaus Hahn (The Scripps Research Institute, CA, USA). The pEGFP-tagged versions of Rab5 (WT), Rab5 (S34N), Rab5 (Q79L), Rab7 (WT), Rab7 (T22N), and Rab7 (Q71L) were kindly provided by Dr. M. Zerial (Max Planck Institute, Dresden, Germany).

### Pharmacological inhibition treatment and viral infection assays

Serum-free medium containing the above inhibitors at specific concentrations was used to pretreat the BHK-21 or PK-15 cells at 37 °C for 30 min. FMDV with multiplicity of infection (MOI) of 1 or 25 was absorbed into the cells at 4 °C for 1 h in the presence of inhibitors. After adsorption, the inoculum was discarded, and unbound virus was removed by rinsing with cold PBS. Subsequently, infection was allowed to proceed at 37 °C by the addition of maintenance medium containing 2% serum, and the inhibitor was maintained throughout the whole infection period. However, MβCD was present only during pretreatment. DMSO-treated cells were incubated in the same conditions and were used as control. After FMDV infection for 1 or 4 h, the cells were fixed and prepared for confocal laser scanning microscopy (CLSM). After FMDV infection for 4 h, infected cells were collected for Western blot and RT-PCR analyses, whereas supernatants were harvested for TCID50 assays.

To determine the effects of drugs on viral entry and post-entry steps, the cells were treated by drugs at 30 min before FMDV addition (MOI 1) or at 1 h after FMDV addition (MOI 1). In the continuous presence of the inhibitors, FMDV infection was allowed at 37 °C. After infection for 4 h, the cells were harvested for Western blot analyses.

### Transmission electron microscopy (TEM)

Monolayers of BHK-21 cells grown on tissue culture plates were infected with FMDV (MOI 100). After 1 h adsorption at 4 °C, the samples were shifted to 37 °C for 5 min. Cell were washed three times with PBS, and cell scarpers were used for the mechanical harvesting of cells. Cell cultures were collected in 1.5 ml Eppendorf tubes and centrifuged at 1000rpm for 5min. The cells pellets were fixed in 2.5% glutaraldehyde at room temperature for 4 h, washed three times in PBS, postfixed with 1% osmium tetroxide for 1 h at 4 °C and washed three times in PBS. Then, samples were dehydrated with gradient ethanol from 50 to 100% (concentrations used were 50, 70, 80, 90, 95 and 100%) for 20min every time and then embedded in epoxy resin. Ultrathin sections were then obtained using an ultramicrotome (Reichert-Jung, Heidelberg, Germany) and double stained by uranyl acetate and lead citrate for 15min at room temperature, respectively. Finally, the sections were examined at 120 keV under a JEM-1010 TEM (JEOL, Tokyo, Japan).

### Confocal laser scanning microscopy (CLSM)

BHK-21 cells were grown on coverslips to subconfluency (~60%). At indicated post-infection (MOI 25), cells were fixed with 4% paraformaldehyde for 20 min and then permeabilized with 0.1% Triton X-100 for 15 min at room temperature. Cells were incubated with 5% new bovine serum (NBS) and incubated with appropriate primary antibodies for 1 h at 37 °C, followed by FITC-, Texas Red-, or TRITC-conjugated secondary antibodies simultaneously. To label actin filaments, cells were incubated with TRITC-phalloidin (2 μg/ml) for 40 min at RT. Nuclei were stained with DAPI. Samples were analyzed by CLSM (Leica SP8) with a 100 × oil immersion objective. The captured images were adjusted for contrast and brightness with Adobe Photoshop software.

To quantify the FMDV entry signal, the CLSM images were acquired randomly and imported to Image J2* software. Then, the number of virus particles inside the cells was automatically measured with a Macro algorithm (developed by CBMSO Confocal Microscopy Service, Spain) in which threshold Intermodes was used to define a single virus particle in the cell and analyzed in 10 individual Mock-, DMSO- or specifical inhibitor-treated cells. Each experiment was performed in triplicate.

### TF, CTxB, and dextran uptake

BHK-21 cells were serum starved for 30 min and then incubated with 10μg/ml Alexa Fluor 594-TF for 15 min at 37 °C. To remove surface-bound TF, an acid buffer (0.2 M acetic acid, 0.5 M NaCl, pH 2.5) was used to wash the cells twice at 4 °C for 5 min each time. After rinsing twice with PBS, the cells were fixed in 4% paraformaldehyde. For CTxB uptake assay, the cells were incubated with 20μg/ml Alexa Fluor 594-CTxB for 45 min at 37 °C. The cells were fixed after rinsing twice with PBS. Finally, the status of TF and CTxB internalization was examined under CLSM.

In accordance with the aforementioned procedures, BHK cells were treated with DMSO or EIPA and then infected with FMDV (MOI 10) for 1 h at 4 °C. Alternatively, the cells were treated with 200 nM PMA as a positive control for 30 min at 37 °C. The cells were then incubated with 0.5 mg/ml Alexa Fluor 594-dextran 10K for 15 min at 37 °C. Dextran uptake was stopped by placing the cells on ice, and the surface-bound dextran was bleached with low pH buffer (0.1 M sodium acetate, 0.05 M NaCl, pH 5.5). The cells were then prepared for CLSM or flow cytometry (BD Bioscience, Canto II).

### Western blot analysis

Cell lysates were generated by adding 1 × SDS-PAGE sample buffer to cells. Samples were boiled for 10 min and fractionated by SDS-PAGE. Proteins were then electrophoretically transferred to polyvinylidene difluoride membranes (Amersham). Membranes were blocked in 5% nonfat milk and incubated with primary antibodies, followed by HRP-conjugated secondary antibodies. Bound antibodies were detected with ECL Plus Western blot detection reagents (PerkinElmer Life Sciences). In all instances, Fig.s are representative of three independent experiments.

### RT-PCR analysis

FMDV-infected BHK-21 cells were collected after 4 hpi, and total RNA was extracted using TRIzol reagent (Ambion) in accordance with the manufacturer’s protocol. Reverse primers for FMDV 3D or β-actin were used to synthesize cDNA fragments using reverse transcriptase M-MLV (TaKaRa). Subsequently, PCR was performed using *rTaq* polymerase (TaKaRa) and specific primers for either FMDV 3D or β-actin (FMDV 3D primers, forward: 5-TTCGGCCTTTGATGCTAACCACT G-3, reverse: 5-GCATCCCGCCCTCAACAACAAT-3; β-actin primers, forward: 5-CGGCATCCACGAAACTAC-3, reverse: 5-ATCTTCATCGTGCTGGGCG-3). For replication of the FMDV 3D gene or β-actin gene, the amplification program was set at 94 °C for 3 min; 18 cycles of 94 °C for 25 s, 56 °C for 25 s, 72 °C for 20 s; and 72 °C for 3 min. The sizes and uniqueness of PCR products were verified by agarose gel electrophoresis. Each test was performed in triplicate.

### TCID50 assay

Collected supernatants were centrifuged to remove cell fragments. Serial tenfold dilutions of virus stock were prepared in serum-free DMEM and added into 96-well plates. Eight replicates were set for each gradient, and 100 μl of virus diluent was added into each well. Subsequently, 100 μl of BHK-21 cell suspension in DMEM with 10% FBS was added into each well at 1.5 × 10^6 ^cells/ml and mixed wells. The cells were incubated at 37 °C under 5% CO_2 _for about 60 h, and the number of wells with or without cytopathic effect (CPE) was counted. The TCID50/100 μl values were calculated by the Reed–Muench method. Each test was performed in triplicate.

### Rac1 activation assays

After serum starvation for 1 h, BHK-21 cells were infected with FMDV (MOI 10). After adsorption for 1 h at 4 °C, the cells were washed with cold PBS, shifted to 37 °C, and collected at indicated time points. Rac1 activation was then detected with the Active Rac1 Detection Kit (Kit #8815 Cell Signaling Technology, Inc.) in accordance with the manufacturer’s recommendations. In brief, GST-PAK1-PBD fusion protein was used to bind the activated form of GTP-bound Rac1, which can then be immunoprecipitated with glutathione resin. Rac1 activation levels were determined using Rac1 mouse mAb.

### siRNA treatment

Liposome RNAiMAX (Invitrogen) was used to mix the specific or nonspecific siRNA with cell suspension, which was coated on six-well plates. After incubation at 37 °C under 5% CO_2_ for 36 h, the cells were infected with FMDV (MOI 1) at 37 °C for 1 h. The cells were then washed with PBS, and fresh medium was added for further incubation for 3 h at 37 °C. The cells were collected and detected by Western blot assay. For each test, Western blot assay was performed to determine the silencing efficiency of CHC and Cav-1. Each interference assay was performed in triplicate.

### Transfection assays

In accordance with the manufacturer’s instructions, BHK-21 cells were transfected with 3 μg/well of specific expression plasmids using Lipofectamine Plus Reagent (Invitrogen) when the cells grew to about 80% confluence. After the cells were incubated in serum-free medium at 37 °C for 6 h, the DNA-reagent mixture was discarded, and incubation continued for 24 h at 37 °C. The cells were then infected with FMDV (MOI 1). After 4 hpi, the cells were collected for Western blot assay.

### Grey scale and statistical analyses

Grey values were quantified using Image J2* software for the bands obtained by Western blot and RT-PCR. All data were normalized against the mean of Mock, DMSO or Control group from at least three independent experiments. Then all data (following a normal distribution) were analyzed using an independent sample *t*-test and expressed as the mean ± standard (SD) of at least three independent samples. P < 0.05 was set as statistically significant, whereas P < 0.01 was considered extremely significant.

## Additional Information

**How to cite this article**: Han, S.-C. *et al.* Productive Entry of Foot-and-Mouth Disease Virus via Macropinocytosis Independent of Phosphatidylinositol 3-Kinase. *Sci. Rep.* **6**, 19294; doi: 10.1038/srep19294 (2016).

## Supplementary Material

Supplementary Information
