# Supplementary material for: Productive Entry of Foot-and-Mouth Disease Virus via Macropinocytosis Independent of Phosphatidylinositol 3-Kinase
Source: Sci Rep. 2016 Jan 13;6:19294. doi: 10.1038/srep19294 (PMC4725844; doi:10.1038/srep19294)
Supplement: Supplementary Information [file srep19294-s1.pdf]

Productive Entry of Foot-and-Mouth Disease Virus via Macropinocytosis Independent of Phosphatidylinositol 3-Kinase

Shi-Chong Han[1†], Hui-Chen Guo[1†], Shi-Qi Sun[1*], Ye Jin[1], Yan-Quan Wei[1], Xia Feng[1], Xue-Ping Yao[2], Sui-Zhong Cao[2], Ding Xing Liu[1,3], Xiang-Tao Liu[1]

Running title: FMDV entry by macropinocytosis

Supplementary Information

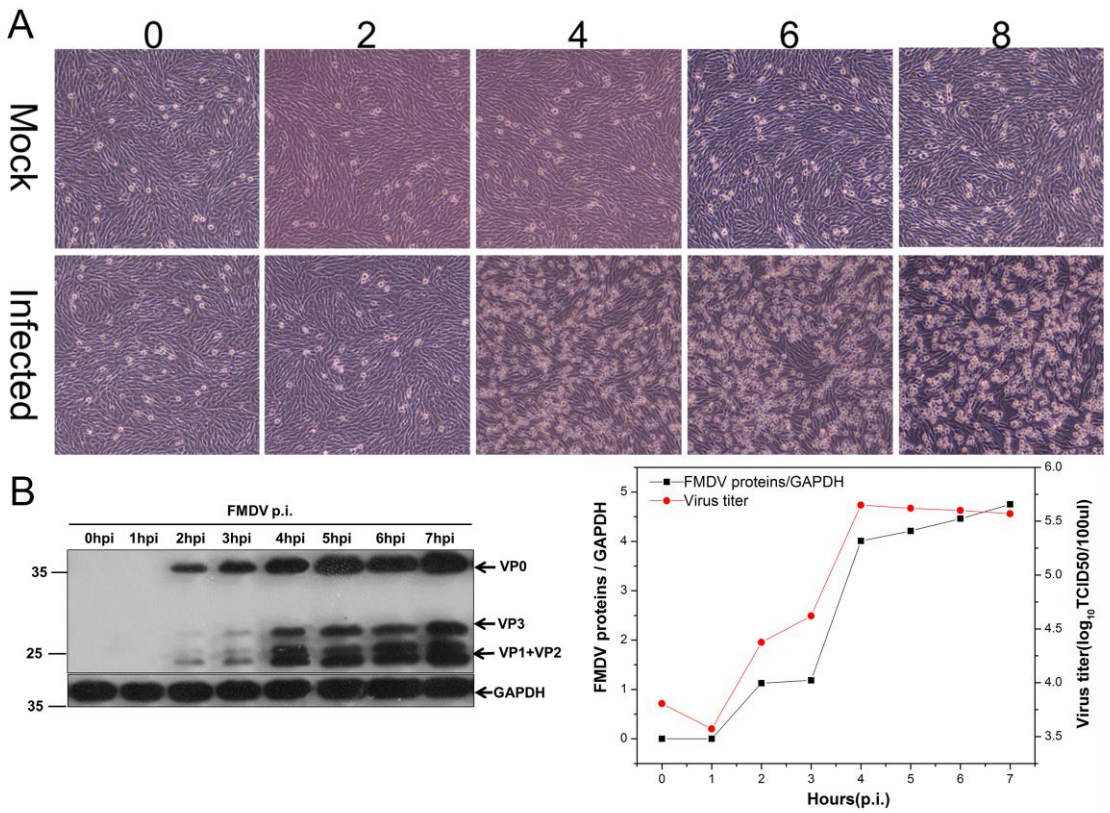

Supplementary Figure 1. Photomicrographs of BHK-21 cells infected with FMDV serotype Asia1 and one-step growth curves of FMDV in PK-15 cells. (A) BHK-21 cells were infected with FMDV (MOI 1; bottom) or mock-infected (top) for the indicated hours post-infection (hpi). Images were taken at an original magnification of 100×. (B) FMDV (MOI 1) was bound to PK-15 cells at 4 ℃.

Unbound virus was removed, and the cells were transferred to 37 ℃. At the indicated time points, the FMDV-infected cells were collected for immunoblotting with an anti-FMDV antibody to determine the amount of FMDV capsid proteins. GAPDH was detected as a load control. The relative quantification of the viral proteins was determined by densitometry as shown in the black line of the graph (right). The FMDV-infected cells were removed to −70 °C, and samples were repeatedly frozen and thawed three times. Virus titers were determined by TCID50 on PK-15 cells as shown in the red line of the graph (right).

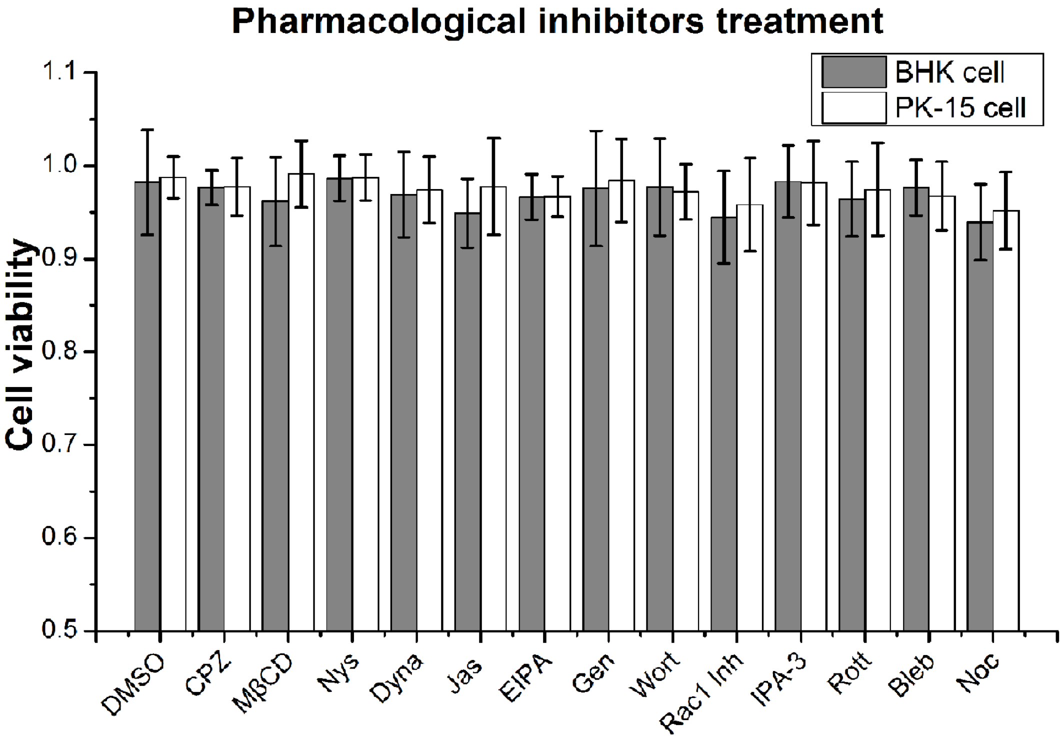

Supplementary Figure 2. Cytotoxicity of pharmacological inhibitors. BHK-21 and PK-15 cells were treated with the maximum concentrations of different pharmacological inhibitors used in this study for 4 h at 37 ℃. Cells were then incubated with Cell Titer 96® Aqueous One Solution Reagent (Promega) for 1 h at 37 ℃, and cell viability was assessed by an enzyme-linked immunosorbent assay plate reader (Bio-Rad) at a test wavelength of 490 nm.

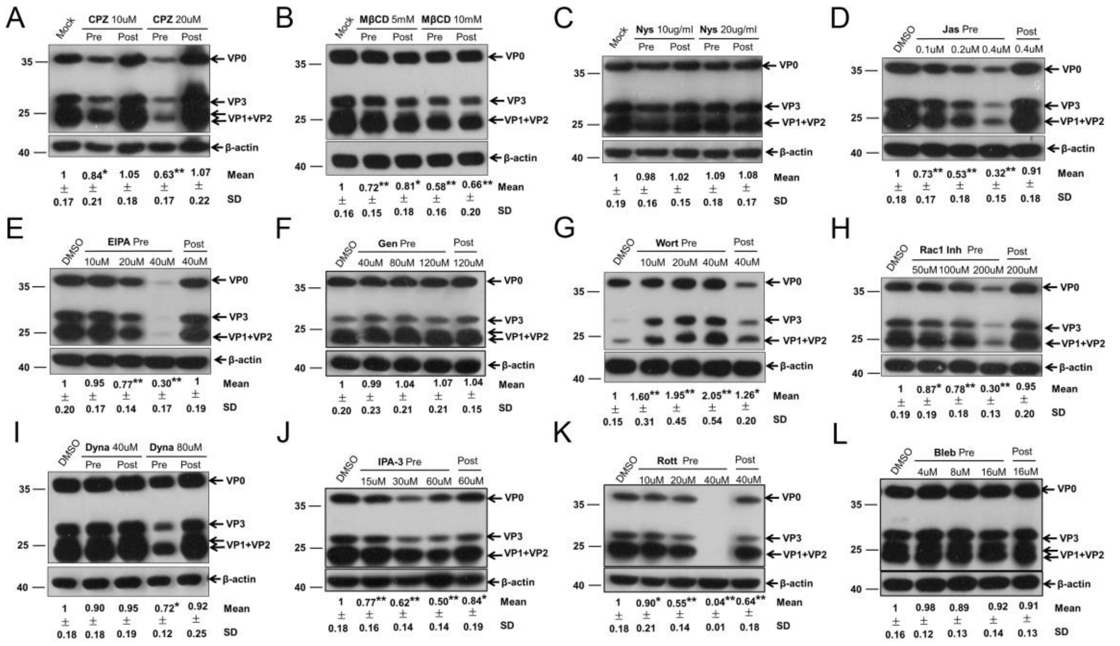

Supplementary Figure 3. Effect of pharmacological inhibitors on FMDV internalization and replication into PK-15 cells. PK-15 cells were treated with various inhibitors 30 min before the infection (Pre) or treated 60 min after virus addition (Post) and maintained during the infection. While MβCD was present only during pretreatment. Cells were then infected (MOI 1) for 4 h at 37 ℃ and analyzed with an anti-FMDV antibody by Western blot, and β-actin was measured as the control. The relative quantification of the viral proteins was determined by densitometry. *, P < 0.05; **, P < 0.01.

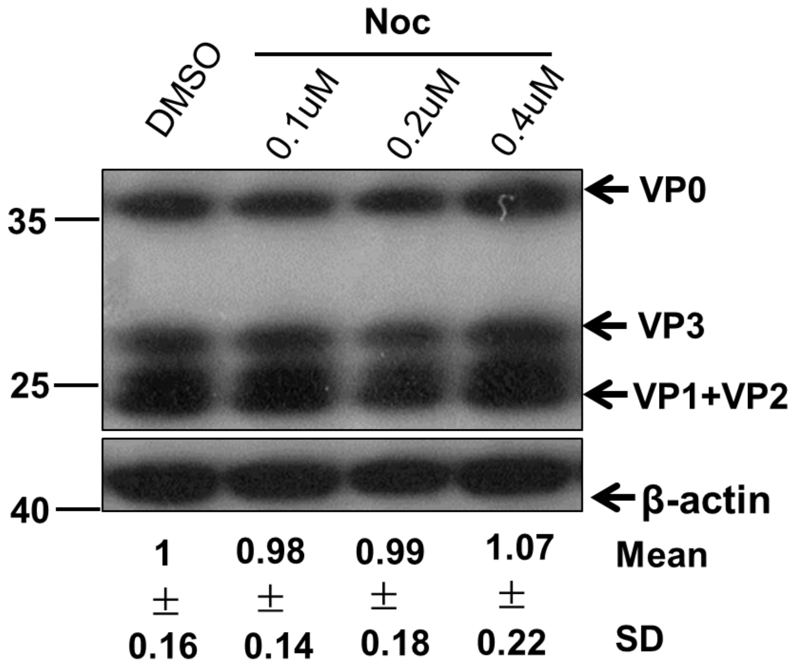

Supplementary Figure 4. Noc does not affect FMDV infection into BHK-21 cells. Cells were pretreated with Bleb 30 min before the infection and maintained during the infection. Cells were then infected (MOI 1) for 4 h at 37 ℃, and analyzed with an anti-FMDV antibody in Western blots. β-Actin was measured as the internal control. The relative quantification of the viral proteins was determined by densitometry. *, P < 0.05; **, P < 0.01.

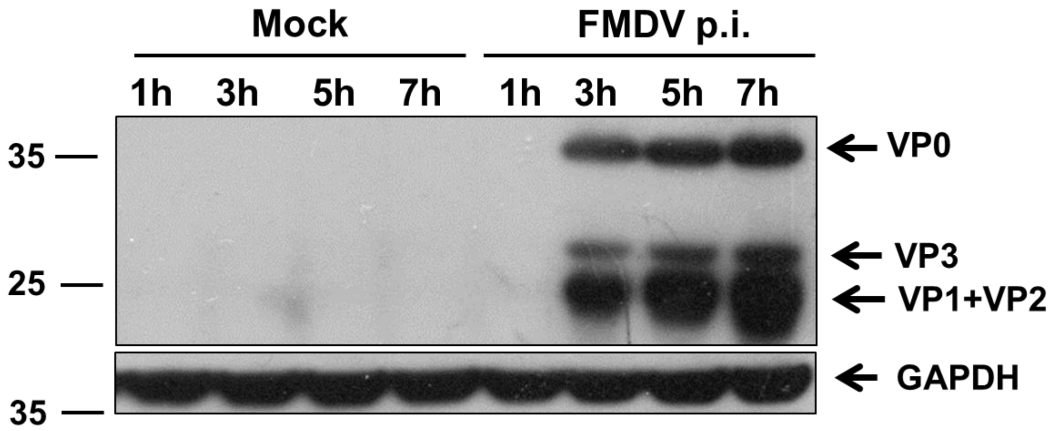

Supplementary Figure 5. Western blot analysis of the specifity of the polyclonal pig antiserum against FMDV. BHK-21 cells were infected with FMDV (MOI 1) or mock-infected. At the indicated time points, the mock-infected and FMDV-infected cells were collected for immunoblotting with this home-made antibody. GAPDH was detected as a load control.
